# Screening of wild species and transcriptome profiling to identify differentially regulated genes in response to late blight resistance in potato

**DOI:** 10.3389/fpls.2023.1212135

**Published:** 2023-07-12

**Authors:** Nisha Bhatia, Jagesh Kumar Tiwari, Chandresh Kumari, Rasna Zinta, Sanjeev Sharma, Ajay Kumar Thakur, Tanuja Buckseth, Dalamu Dalamu, Rajesh Kumar Singh, Vinod Kumar

**Affiliations:** ^1^Division of Crop Improvement, Indian Council of Agricultural Research—Central Potato Research Institute, Shimla, Himachal Pradesh, India; ^2^School of Biotechnology, Shoolini University, Solan, Himachal Pradesh, India; ^3^Division of Vegetable Improvement, Indian Council of Agricultural Research—Indian Institute of Vegetable Research, Varanasi, Uttar Pradesh, India; ^4^School of Bioengineering and Biosciences, Lovely Professional University, Phagwara, Punjab, India

**Keywords:** genes, late blight resistance, potato, screening, *Solanum* species, transcriptome sequencing

## Abstract

Late blight (*Phytophthora infestans*) is a serious disease of potatoes. The aim of this study was to screen wild potato species and identify differentially expressed genes (DEGs) associated with late blight resistance. Wild potato species such as PIN45 (*Solanum pinnatisectum*), CPH62 (*Solanum cardiophyllum*), JAM07 (*Solanum jamesii*), MCD24 (*Solanum microdontum*), PLD47 (*Solanum polyadenium*), and cv. Kufri Bahar (control) were tested by artificial inoculation of *P. infestans* under controlled conditions. Transcriptomes of the leaf tissues (96 h post-inoculation) were sequenced using the Illumina platform. Statistically significant (*p* < 0.05) DEGs were analyzed in wild species by comparison with the control, and upregulated (>2 log_2_ fold change, FC) and downregulated (<−2 log_2_ FC) genes were identified. DEGs were functionally characterized with Gene Ontology (GO) terms and Kyoto Encyclopedia of Genes and Genomes (KEGG) pathways. Selected genes were validated by real-time PCR analysis to confirm RNA-seq results. We identified some upregulated genes associated with late blight resistance in wild species such as cytochrome P450, proline-rich protein, MYB transcription factor MYB139, ankyrin repeat-containing protein, and LRR receptor-like serine/threonine-protein kinase in PIN45; glucosyltransferase, fructose-bisphosphate aldolase, and phytophthora-inhibited protease 1 in CPH62; steroid binding protein and cysteine proteinase 3 in JAM07; glycine-rich cell wall structural protein 1 and RING finger protein in MCD24; and cysteine proteinase 3 and major latex protein in PLD47. On the other hand, downregulated genes in these species were snakin-2 and WRKY transcription factor 3 in PIN45; lichenase and phenylalanine ammonia-lyase 1 in CPH62; metallothionein and LRR receptor-like serine/threonine-protein kinase in JAM07; UDP-glucoronosyl/UDP-glucosyl transferase family protein and steroid binding protein in MCD24; and cytoplasmic small heat shock protein class I and phosphatase PLD47. Our study identified highly resistant wild potato species and underlying genes such as disease resistance, stress response, phytohormones, and transcription factors (e.g., MYB, WRKY, AP2/ERF, and AN1) associated with late blight resistance in wild potato species.

## Introduction

Late blight, caused by the oomycetes *Phytophthora infestans* (Mont.) de Bary, is the most devastating disease of potatoes (Hawkes, 1990). This pathogen is highly variable, which poses problem in its management, and therefore, cultivated potatoes lack a durable resistance source. The genus *Solanum* is one of the richest sources of genetic diversity in plant species, but only a small fraction of diploid wild species has been utilized in potato improvement (Bradshaw et al., 2006). In the past, the race-specific resistance sources have been identified in the hexaploid wild species *Solanum demissum*, and resistance have been deployed in potato breeding. However, the *R* genes were gradually defeated due to the emergence of new strains and new effector molecules of *P. infestans*. Hence, there is a need for new resistance source against *P. infestans*. A number of wild potato species have been found to be highly resistant to late blight such as *Solanum pinnatisectum*, *Solanum cardiophyllum*, *Solanum jamesii*, *Solanum microdontum*, and *Solanum polyadenium* (Tiwari et al., 2015) and characterized at molecular level by simple sequence repeat (SSR) markers and phenotypes (Tiwari et al., 2019). However, these wild species have been utilized at limited scale due to the sexual barriers with common potato (Jansky, 2006). However, resistance have been incorporated from wild species into the cultivated potato via somatic hybridization and other biotechnological tools (Tiwari, 2023). For instance, we have demonstrated the use of *S. pinnatisectum* and *S. cardiophyllum* via somatic hybridization for late blight resistance in potato (Sarkar et al., 2011; Chandel et al., 2015). Furthermore, selected lines of five wild species, namely, PIN45 (*S. pinnatisectum*, CGN 17745), CPH62 (*S. cardiophyllum*, PI 283062), JAM07 (*S. jamesii*, PI 498407), MCD24 (*S. microdontum*, PI 218224), and PLD47 (*S. polyadenium*, CGN 17747) have been registered as elite genetic stocks for their unique traits (late blight resistance and wild species) with the National Active Germplasm Site (NAGS) by the Indian Council of Agricultural Research-National Bureau of Plant Genetic Resources (ICAR-NBPGR), New Delhi, India. However, these wild species have not been characterized at transcripts level for late blight resistance genes.

With the availability of the potato genome sequence (Potato Genome Sequencing Consortium, 2011) and post-genomics advancements (Tiwari, 2023), it is now possible to analyze complete genes information at a whole genome level in potato. Many reports are available on transcriptome analysis in potato for late blight resistance (Gao and Bradeen, 2016; Yang et al., 2018; Yang et al., 2020; Frades et al., 2015) and other abiotic-stress-related N stress (Tiwari et al., 2020). Gao and Bradeen (2016) revealed that that *R* gene and ethylene and stress responsive genes contribute to the *RB* gene-mediated incompatible potato–*P. infestans* interactions in both the foliage and tubers of potato. Various transcriptome-based studies revealed gene networks governing late blight resistance after *P. infestans* infection on host–pathogen interaction. The study provides an overview of compatible and incompatible *P. infestans* interaction in potato *S. tuberosum* Gp. The Andigena genotype (03112-233) is resistant to isolate 90128 but susceptible to the super race isolate CN152 (Duan et al., 2020). Li and Zhao (2021) analyzed the transcriptome in grafted scions onto potato rootstock to improve late blight resistance. In another study, global transcriptome analyses showed the molecular signatures in the early response of potato to *P. infestans*, *Ralstonia solanacearum*, and *Potato virus Y* infection in potato (Cao et al., 2020).

In this study, we aimed to identify genes in response to *P. infestans* resistance by transcriptome sequencing in wild potato species. RNA-seq data was analyzed for differentially expressed genes (DEGs), heat map, Venn diagram, Gene Ontology (GO) annotation, Kyoto Encyclopedia of Genes and Genomes (KEGG) pathways analysis, and potential genes to be involved in late blight resistance in potato. A few selected DEGs were confirmed by real-time quantitative PCR analysis. Our study provides genes and transcription factors involved in late blight resistance in potato. This information can be useful in designing strategies for *P. infestans* management by breeding and genomics approaches.

## Materials and methods

### Plant materials

A total of 40 genotypes were used for late blight resistance test, and the selected five wild species and one control (Kufri Bahar) were used for transcriptome study. Details of the potato genotypes are listed in **Table 1**. A total of 19 wild species, 18 interspecific somatic hybrids, and 3 potato varieties Kufri Girdhari (highly resistant), Kufri Jyoti (susceptible), and Kufri Bahar (highly susceptible, control) were used in late blight test. All the genotypes were used from the germplasm repository available at the Indian Council of Agricultural Research-Central Potato Research Institute, Shimla, Himachal Pradesh, India. The selected five wild species, *viz*., PIN45 (*S. pinnatisectum*, CGN17745), CPH62 (*S. cardiophyllum*, PI283062), JAM07 (*S. jamesii*, PI498407), MCD24 (*S. microdontum*, PI218224), and PLD47 (*S. polyadenium*, CGN17747), and Kufri Bahar (control, highly susceptible) were used for RNA-seq study. The selection of the wild species was based on our previous research (Tiwari et al., 2015) and this study, showing high resistance to late blight. Virus-free *in vitro* plants were maintained at the institute. Plants were multiplied for further research by sub-culturing of leafy nodes on the MS medium (Murashige and Skoog, 1962) at pH 5.8 supplemented with sucrose (20 g/L) and solidified with gelrite (2 g/L), and cultures were grown at 20°C under a 16-h photoperiod (light intensity, 50–60 µmol/m^2^/s) (Sarkar et al., 2011).

**Table 1:** Late blight resistance test of wild (*Solanum*) species, somatic hybrids, and common potato varieties under controlled conditions by artificial inoculation of *Phytophthora infestans*.

Sr. No.	Genotype	*Solanum* species (Acc. ID)	Late blight incidence (AUDPC value) (% day)			Class*
			2021	2022	Mean	
1.	ACL38	*Solanum acaule* (CGN17938)	49.38	61.25	55.32	R
2.	BER57	*S. berthaultii* (PI265857)	0.00	3.50	1.75	HR
3.	CPH62	*S. cardiophyllum* (PI283062)	2.77	10.50	6.63	HR
4.	CPH33	*S. cardiophyllum* (PI341233)	90.50	95.25	92.88	R
5.	CHC60	*S. chacoense* (PI197760)	24.17	28.50	26.33	HR
6.	IOP80	*S. iopetalum* (PI230480)	128.84	130.75	129.79	MR
7.	IOP59	*S. iopetalum* (PI230459)	0.00	0.00	0.00	HR
8.	JAM07	*S. jamesii* (PI498407)	0.00	2.50	1.25	HR
9.	LES29	*S. lesteri* (CGN24429)	138.34	152.75	145.54	MR
10.	MCD24	*S. microdontum* (PI218224)	12.00	18.00	15.00	HR
11.	PNT44	*S. pinnatisectum* (CGN17444)	12.00	13.75	12.88	HR
12.	PNT43	*S. pinnatisectum* (CGN17443)	8.50	18.50	13.50	HR
13.	PIN45	*S. pinnatisectum* (CGN17445)	7.34	10.75	9.04	HR
14.	PLD47	*S. polyadenium* (CGN17747)	0.00	10.50	5.25	HR
15.	PLD48	*S. polyadenium* (CGN17748)	18.67	24.75	21.71	HR
16.	PLT50	*S. polytrichon* (CGN22350)	0.00	0.00	0.00	HR
17.	STO40	*S. stoloniferum* (SS2740)	63.67	81.25	72.46	R
18.	TRF65	*S. trifidum* (PI255565)	0.00	7.50	3.75	HR
19.	VEN30	*S. vernei* (PI320330)	64.17	72.75	68.46	R
20.	P1	*S. tuberosum* (*+*) *S. pinnatisectum*	74.67	88.00	81.33	R
21.	P2	*S. tuberosum* (*+*) *S. pinnatisectum*	77.50	87.50	82.50	R
22.	P3	*S. tuberosum* (*+*) *S. pinnatisectum*	77.50	79.00	78.25	R
23.	P4	*S. tuberosum* (*+*) *S. pinnatisectum*	59.17	67.50	63.33	R
24.	P5	*S. tuberosum* (*+*) *S. pinnatisectum*	69.17	73.50	71.33	R
25.	P6	*S. tuberosum* (*+*) *S. pinnatisectum*	52.34	72.00	62.17	R
26.	P7	*S. tuberosum* (*+*) *S. pinnatisectum*	47.84	55.50	51.67	R
27.	P8	*S. tuberosum* (*+*) *S. pinnatisectum*	57.50	68.75	63.13	R
28.	P9	*S. tuberosum* (*+*) *S. pinnatisectum*	50.84	70.25	60.54	R
29.	P10	*S. tuberosum* (*+*) *S. pinnatisectum*	31.34	43.75	37.54	HR
30.	P11	*S. tuberosum* (*+*) *S. pinnatisectum*	0.00	0.00	0.00	HR
31.	P12	*S. tuberosum* (*+*) *S. pinnatisectum*	0.00	9.25	4.63	HR
32.	P13	*S. tuberosum* (*+*) *S. pinnatisectum*	6.84	14.25	10.54	HR
33.	P14	*S. tuberosum* (*+*) *S. pinnatisectum*	6.00	3.50	4.75	HR
34.	Crd6	*S. tuberosum* (+) *S. cardiophyllum*	0.00	5.50	2.75	HR
35.	Crd10	*S. tuberosum* (+) *S. cardiophyllum*	2.17	7.00	4.58	HR
36.	Crd16	*S. tuberosum* (+) *S. cardiophyllum*	1.17	3.00	2.08	HR
37.	Crd23	*S. tuberosum* (+) *S. cardiophyllum*	33.34	38.25	35.79	HR
38.	Kufri Jyoti	*S. tuberosum* Gp. Tuberosum	156.34	178.75	167.54	S
39.	Kufri Girdhari	*S. tuberosum* Gp. Tuberosum	0.00	2.25	1.13	HR
40.	Kufri Bahar	*S. tuberosum* Gp. Tuberosum	196.67	218.00	207.33	S
CD (*p* < 0.05)			8.68	9.55	13.11	

*Genotypes were classified based on the area under disease progressive curve (AUDPC) value: highly resistant (HR ≤ 50), resistant (R = 50–100), moderately resistant (MR = 100–150), and susceptible (S ≥ 150).

### Late blight resistance test

*In vitro* plants were grown in the earthen pots (20×25 cm^2^) with three biological replications containing a sterile mixture of soil/FYM-based compost (1:1, v/v) under a glasshouse during the summer season (31.10°N, 7.17°E, 2,200 m above mean sea level) at the institute following standard practices (Tiwari et al., 2015). All genotypes were challenge inoculated by the fungus pathogen *P. infestans* isolate HP09/40 (A2 mating type and races 1.2.3.4.5.6.7.8.9.10.11) for late blight resistance test under artificially controlled conditions (18 ± 2°C temperature, and 80%–90% relative humidity) (Tiwari et al., 2015). The area under disease progressive curve (AUDPC) was calculated based on the percent disease infestation on leaves or whole plants (Shaner and Finney, 1977), and accordingly, genotypes were classified based on the AUDPC value (% day) as highly resistant (HR ≤ 50), resistant (R = 50–100), moderately resistant (MR = 100–150), and susceptible (S ≥ 150) (Singh and Bhattacharyya, 1995). Leaf tissues were collected from the selected six samples at 96-h post-inoculation stage of *P. infestans*. Samples were snap-frozen in liquid nitrogen and stored at −80°C until further use. The tissue from three biological replicates was pooled together to make a single sample, and transcriptome analysis was performed in two technical replicates.

### Total RNA sequencing and reference potato mapping

Total RNA sequencing and analysis was performed following our earlier procedures (Tiwari et al., 2020). Briefly, total RNA was isolated from the leaf tissues of six samples (each with three biological replications) using a modified c-TAB and lithium chloride method (Rubio-Pifia and Zapata-Peter, 2011). The paired-end (PE) sequencing libraries were prepared following the manufacturer’s instructions (Illumina, San Diego, CA, USA). RNA-seq was performed in two technical replicates. The PCR-enriched libraries were analyzed on 4200 Tape Station system (Agilent Technologies, Santa Clara, CA, USA). The PE Illumina libraries were sequenced in Illumina NovaSeq 6000. The raw data were processed to obtain high-quality clean reads using Trimmomatic v0.38 to remove adapter sequences and ambiguous reads. The high-quality (QV>25) paired-end reads were used for reference mapping with the potato genome of *S. tuberosum* Group Phureja DM1-3 using TopHat v2.1.1 with default parameters (Trapnell et al., 2009).

### Differential gene expression, Venn diagram, and heat map analysis

Transcriptome data were assembled using cufflinks program, and then, data were used to identify differentially expressed genes (DEGs) using the software cuffdiff version 2.2.1 (Trapnell et al., 2013). DEGs were analyzed in all five highly resistant wild species, namely, PIN45, CPH62, JAM07, MCD24, and PLD47 using Kufri Bahar (highly susceptible) as a control. Fragments per kilobase of transcript per million mapped reads (FPKM) value was used to calculate the log_2_ fold change (FC). The log_2_ FC value >0 were considered upregulated, whereas the log_2_ FC value <0 were downregulated along with *p*-value threshold of 0.05 for statistically significant results. Significant DEGs were identified based on the statistical significance (*p* ≤ 0.05) for upregulated genes (≥2.0 log_2_ FC) and downregulated genes (<−2.0 log_2_ FC). Venn diagrams of up- and downregulated DEGs were identified using Venny 2.1 tool (Oliveros, 2007-2015). An average linkage hierarchical cluster analysis was performed with the top 50 DEGs (25 each of up- and downregulated) using multiple experiments viewer (MeV v4.9.0) (Howe et al., 2011) following our earlier procedures (Tiwari et al., 2020).

### GO and KEGG pathways analysis

The GO annotations of the DEGs were obtained from the Ensembl Plants database for *S. tuberosum*. The GO annotations were categorized into upregulated, downregulated, and expressed in both and exclusive DEGs. The information on the number of genes was assigned into three main GO domains (biological process, cellular component, and molecular function). The bar plots depicting the GO distribution were obtained through the WEGO portal (http://wego.genomics.org.cn/cgi-bin/wego/index.pl) (Ye et al., 2006). The functional annotations of the DEGs were carried out against the curated KEGG GENES database using KAAS [KEGG Automatic Annotation Server (http://www.genome.jp/kegg/ko.html)] (Moriya et al., 2007). The KEGG orthology (KO) database of Nightshade family was used as the reference for pathways mapping.

### KEGG pathway enrichment analysis

KEGG pathway enrichment analysis of DEGs was performed to gain insights into the underlying biology of differentially expressed genes and proteins. Analyzing high-throughput molecular measurements at the functional level is very appealing for two reasons. First, grouping thousands of genes, proteins, and/or other biological molecules by the pathways they are involved in reduces the complexity to just several hundred pathways for the experiment. Second, identifying active pathways that differ between two conditions can have more explanatory power than a simple list of different genes or proteins. ClusterProfiler was used for the pathway enrichment at a *p*-value cutoff of 0.05. ClusterProfiler, an R Bioconductor package, was used for KEGG pathway analysis using the information of the reference genome. Furthermore, a KEGG mapper (https://www.genome.jp/kegg/mapper/) was used to search mapping information about the genes involved in plant–pathogen interaction.

### Real-time quantitative polymerase chain reaction analysis

Selected 10 DEGs were validated through RT-qPCR analysis following our earlier procedures (Tiwari et al., 2020). The RT-qPCR primers were designed using IDT PrimerQuest Tool (https://eu.idtdna.com/Primerquest/Home/Index). RT-PCR analysis was performed using Power SYBR Green PCR Master Mix in ABI PRISM HT7900 (Applied Biosystems Warrington, UK) following temperature/time profile 50°C for 2 min; 95°C for 10 min; and 40 cycles of 95°C for 15 s, 60°C for 1 min, and 72°C for 30 s with an internal standard potato ubiquitin-ribosomal protein gene (*ubi3*; L22576).

## Results

### Late blight resistance assay

**Figure 1:**
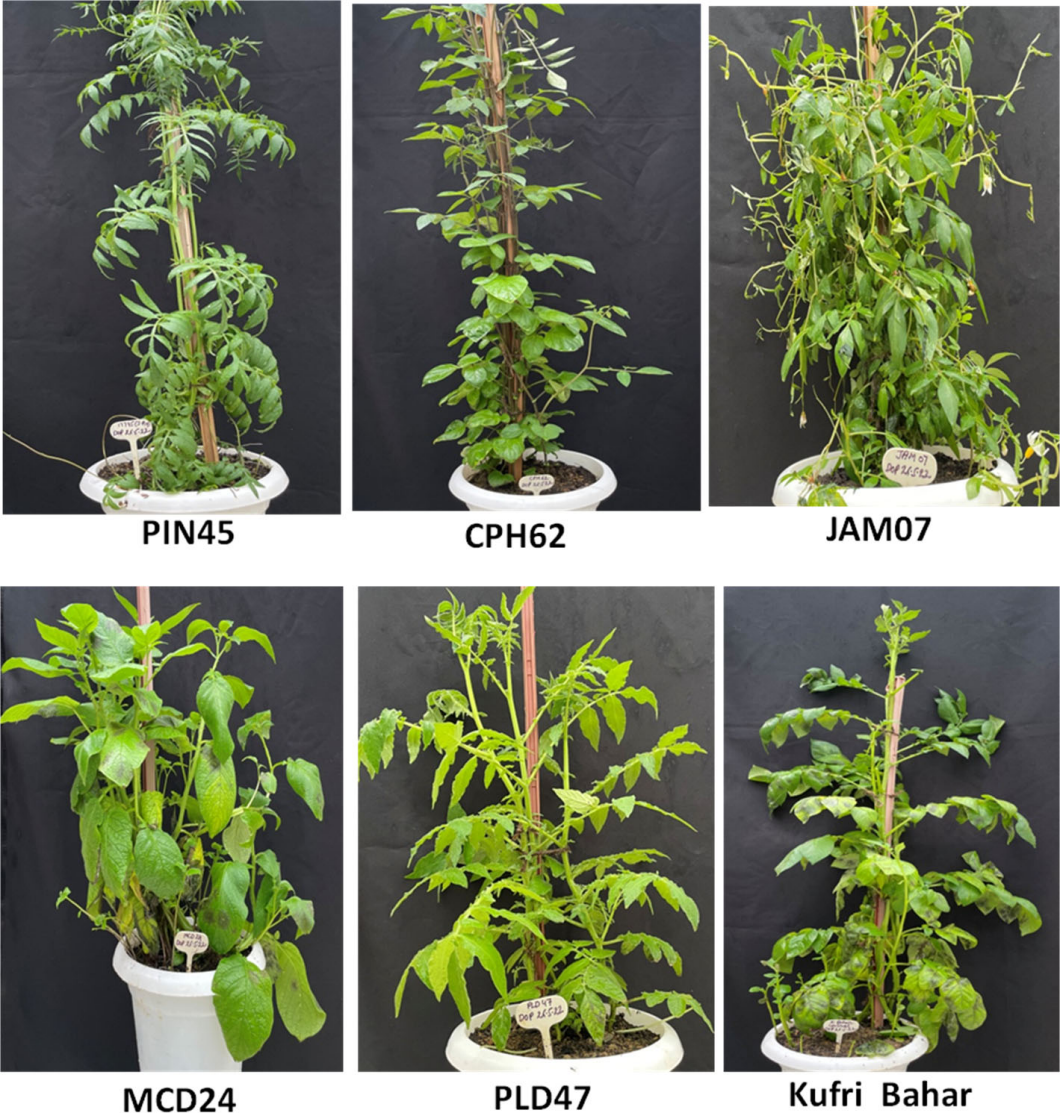
Late blight resistance assay of potato wild species, *viz.*, PIN45 (*Solanum pinnatisectum*, CGN 17745), CPH62 (*S. cardiophyllum*, PI 283062), JAM07 (*S. jamesii*, PI 498407), MCD24 (*S. microdontum*, PI 218224), PLD47 (*S. polyadenium*, CGN 17747), and control variety Kufri Bahar (highly susceptible) by artificial inoculation of *Phytophthora infestans*.

A total of 40 potato genotypes including wild species, interspecific somatic hybrids, and common potato varieties were tested for late blight resistance in 2 years (2021 and 2022) under artificial controlled conditions by challenge inoculation of *P. infestans* (**Figure 1**). Resistance class was categorized based on the AUDPC values calculated based on the percent disease infection on leaves and stems. Based on 2 years screening data, 23 genotypes were highly resistant, 2 moderately resistant, 13 resistant, and 2 susceptible (**Table 1**). Among all, five highly resistant wild species were selected for further transcriptome analysis, i.e., PIN45 (AUPDC = 9.04), CPH62 (AUPDC = 6.63), JAM07 (AUPDC = 1.25), MCD24 (AUPDC = 15.00), and PLD47 (5.25), whereas control Kufri Bahar (AUPDC = 207.33) was found to be highly susceptible.

### RNA-seq data generation and reference mapping

High-quality paired-end read data (QV > 25) were generated in two technical replications of RNA-sequencing data in the leaf tissues of samples such as PIN45 (9.73/8.43 Gb), CPH62 (11.05/9.51 Gb), JAM07 (9.03/10.36 Gb), PLD47 (7.29/6.83 Gb), MCD24 (9.25/11.26 Gb), and Kufri Bahar (8.44/7.95 Gb). Furthermore, reference mapping of the reads revealed mapping results such as CPH62 (67.3/62.4%), PIN45 (63.6/72.4%), JAM07 (70.4/70.7%), PLD47 (57.6/56.8%), MCD24 (82.1/83.8%), and Kufri Bahar (61.2/58.6%).

### Identification of differentially expressed genes

**Figure 2:**
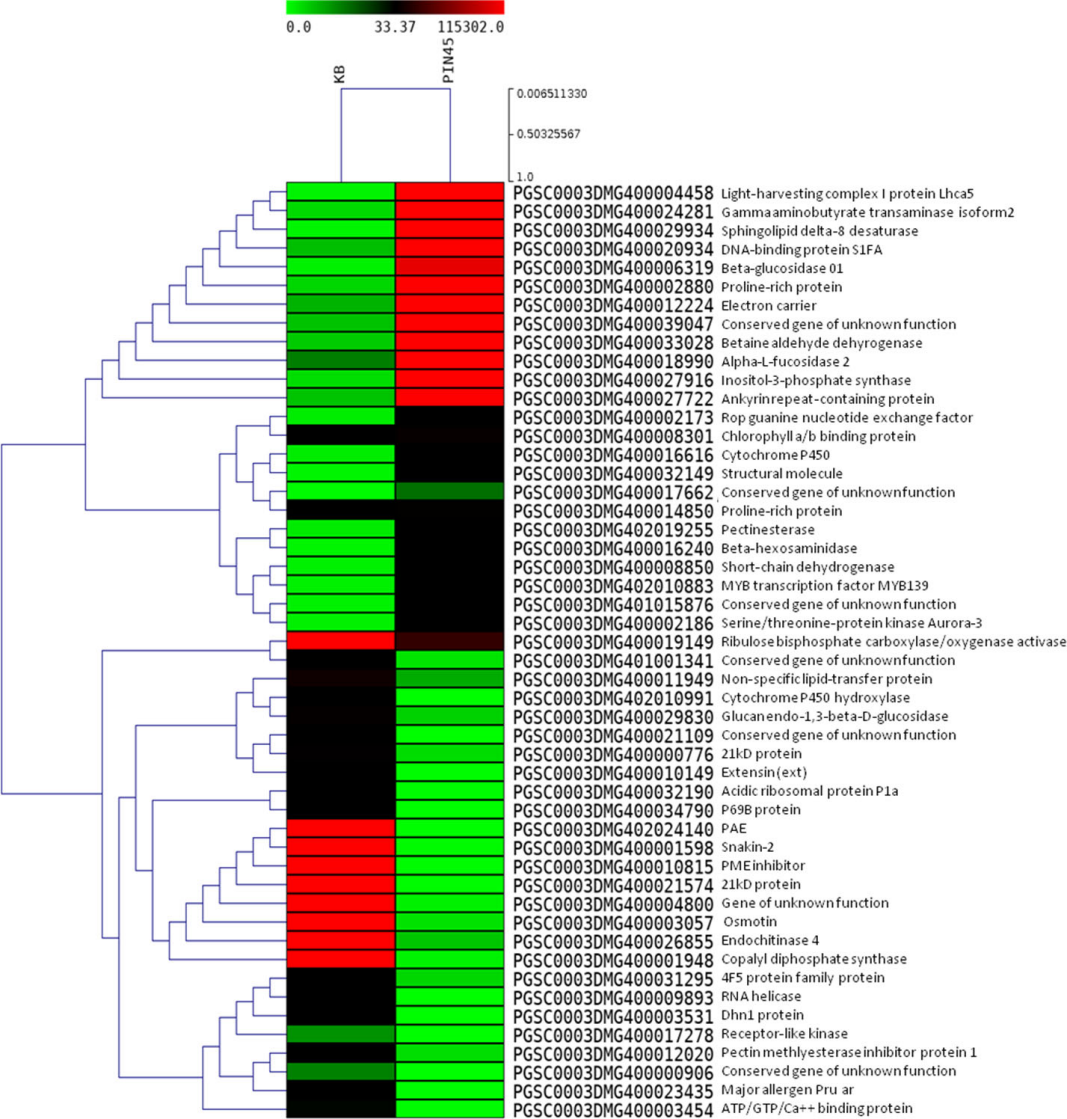
Heat maps of top 50 differentially expressed genes in potato wild species PIN45 (*S. pinnatisectum*) versus Kufri Bahar (control) analyzed by RNA-seq in leaf tissues after artificial inoculation of *P. infestans* under controlled conditions. In the heat maps, each horizontal line refers to a gene. Relatively upregulated genes are shown in red color, whereas downregulated genes are shown in green color.

**Figure 3:**
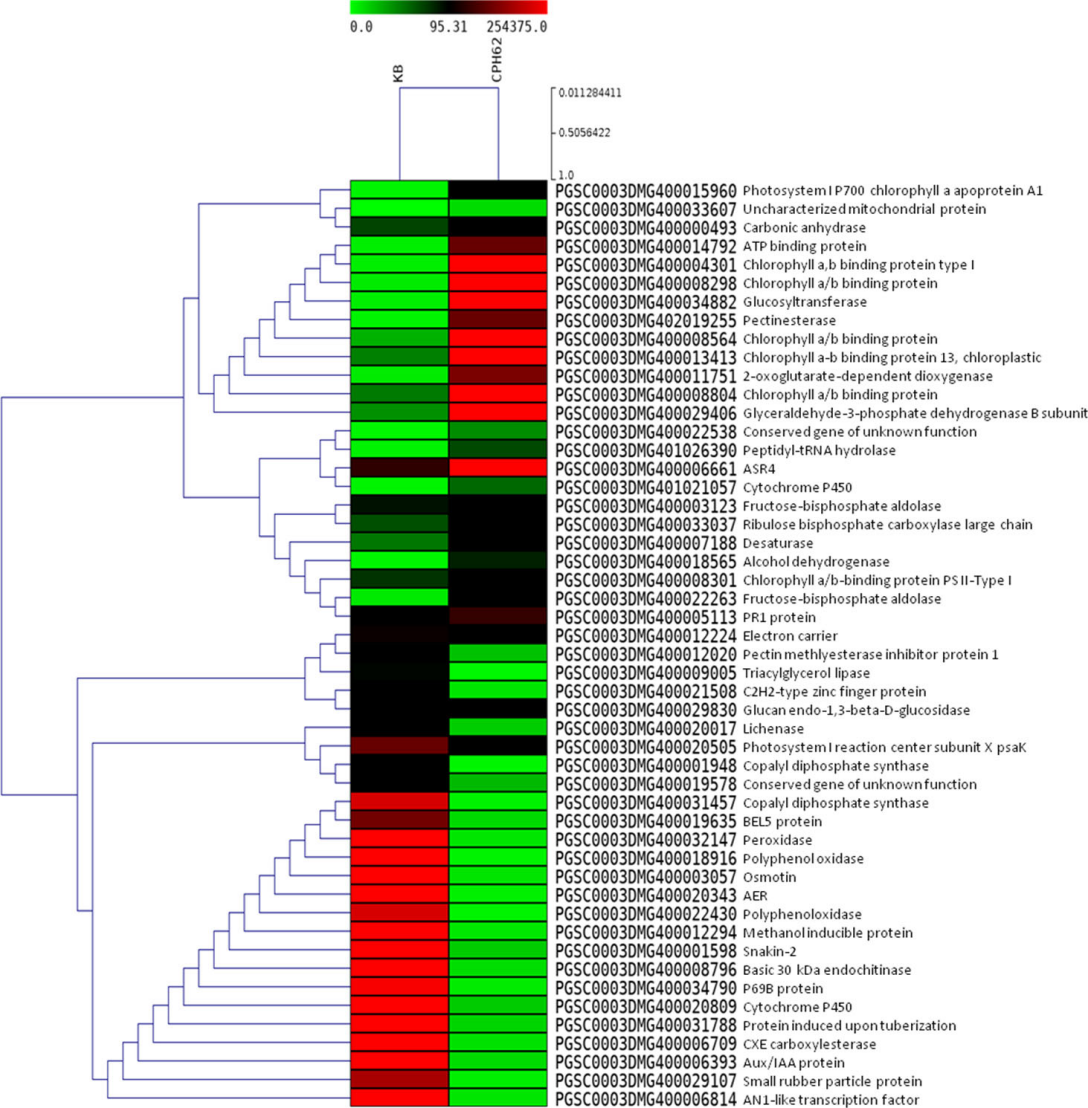
Heat maps of top 50 differentially expressed genes in potato wild species CPH62 (*S. cardiophyllum*) versus Kufri Bahar (control) analyzed by RNA-seq in leaf tissues after artificial inoculation of *P. infestans* under controlled conditions. In the heat maps, each horizontal line refers to a gene. Relatively upregulated genes are shown in red color, whereas downregulated genes are shown in green color.

**Figure 4:**
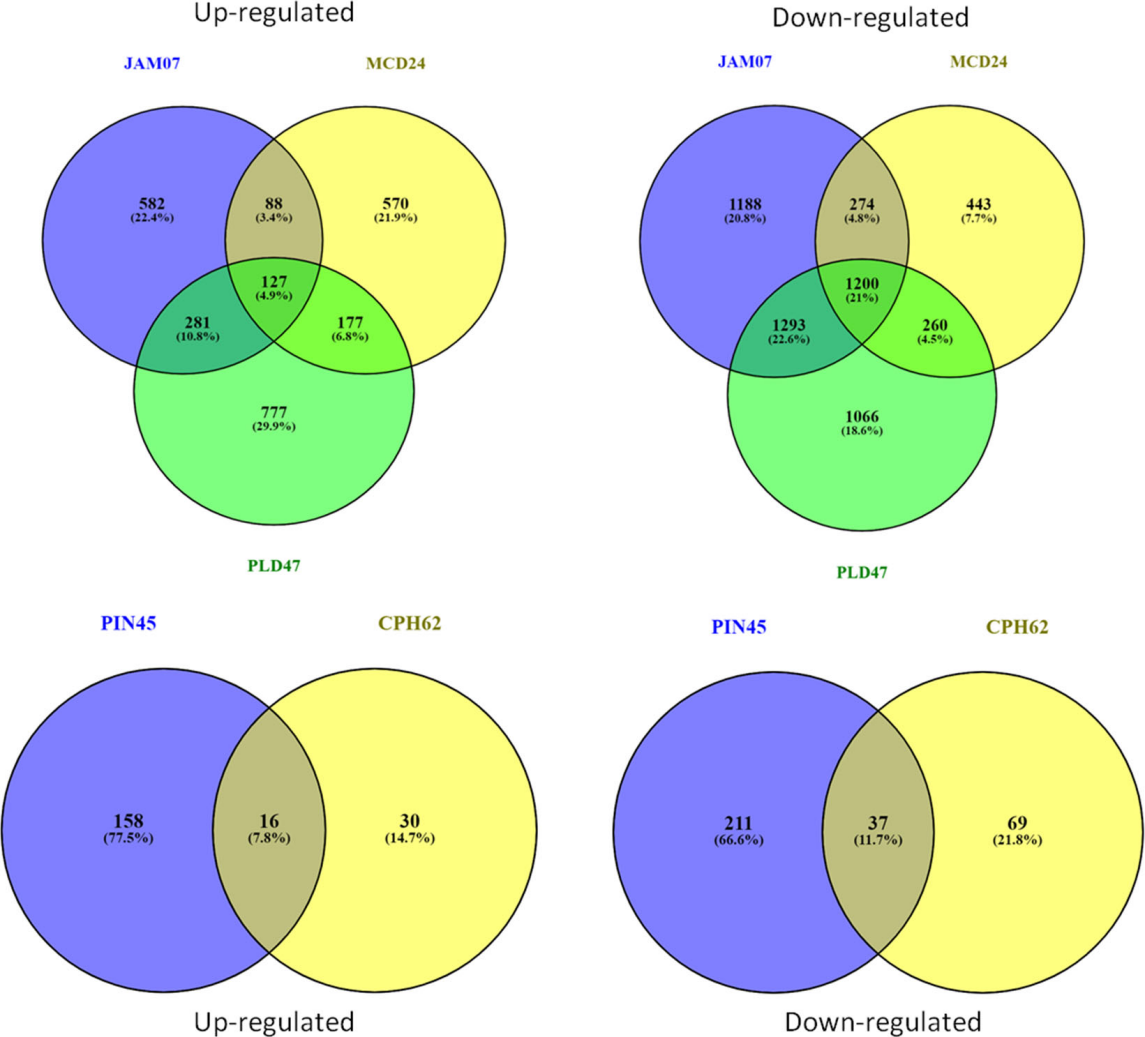
Venn diagrams showing common genes (upregulated/downregulated) among wild potato species, *viz.*, PIN45 (*S. pinnatisectum*), CPH62 (*S. cardiophyllum*), JAM07 (*Solanum jamesii*), MCD24 (*S. microdontum*), and PLD47 (*S. polyadenium*).

Statistically significant (*p* < 0.05) upregulated (≥ 2.0 log_2_ FC) and downregulated (<−2.0 log_2_ FC) DEGs were identified in the wild species versus susceptible control Kufri Bahar. The selected top 20 DEGs (up- and downregulated) are summarized in **Table 2**. The gene expression (log_2_ FC) varied differently such as PIN445 (upregulated, 2.40–7.04; downregulated, −11.00 to −2.77), CPH62 (upregulated, 2.59–6.13; downregulated, −6.48 to −2.54). In JAM07, out of a total of 7,884 DEGs, statistically significant DEGs upregulated were 1,078 (13.98 to 2.00 log_2_ FC) and downregulated were 3,955 (−15.31 to −2.00 log_2_ FC). Of the total of 4,928 DEGs in MCD24, statistically significant upregulated were 962 (16.73 to 2.00 log_2_ FC) and downregulated were 2,177 (−15.29 to −2.00 log_2_ FC). In PLD47 (vs. KB), of a total of 8,094 DEGs, statistically significant upregulated DEGs were 1,362 (12.36 to 2.00 log_2_ FC) and downregulated were 3,819 (−14.01 to −2.00 log_2_ FC). Heat maps of selected 50 genes are shown in **Figure 2** (PIN45) and **Figure 3** (CPH62). Remaining heat maps are given in **Supplementary Files** in **Supplementary Figure S1** (JAM07), **Supplementary Figure S2** (MCD24), and **Supplementary Figure S3** (PLD47). Common genes were identified by Venn diagram analysis. A total of 127 upregulated genes and 1,200 downregulated genes were common among JAM07, MCD24, and PLD47, whereas only 16 upregulated and 37 downregulated genes were common between PIN45 and CPH62 (**Figure 4**). DEGs are given in **Supplementary Excel Files** (**Supplementary Datasets 1–5**).

**Table 2:** Selected differentially expressed genes (DEGs) (*p* < 0.05) in response to *P. infestans* infection in the leaf tissues of wild potato species.

Sr. No.	Gene ID	Locus	Gene description	Gene expression* (Log_2_ FC)
i) PNT45				
Upregulated				
1.	PGSC0003DMG400004458	Chr_ST4.03ch07:31603458-31605657	Light-harvesting complex I protein Lhca5	7.046
2.	PGSC0003DMG400024281	Chr_ST4.03ch12:5776515-5780741	Gamma aminobutyrate transaminase isoform2	6.716
3.	PGSC0003DMG400029934	Chr_ST4.03ch08:35730231-35732357	Sphingolipid delta-8 desaturase	6.602
4.	PGSC0003DMG400006319	Chr_ST4.03ch01:66544366-66552432	Beta-glucosidase 01	5.909
5.	PGSC0003DMG400002173	Chr_ST4.03ch09:1075675-1079306	Rop guanine nucleotide exchange factor	5.751
6.	PGSC0003DMG400016616	Chr_ST4.03ch09:60536375-60538029	Cytochrome P450	5.596
7.	PGSC0003DMG400002880	Chr_ST4.03ch12:2714288-2715753	Proline-rich protein	5.588
8.	PGSC0003DMG402010883	Chr_ST4.03ch06:5965435-5966610	MYB transcription factor MYB139	4.854
9.	PGSC0003DMG400027722	Chr_ST4.03ch03:35562832-35565426	Ankyrin repeat-containing protein	4.834
10.	PGSC0003DMG400022264	Chr_ST4.03ch07:55852619-55856340	LRR receptor-like serine/threonine-protein kinase	3.869
Downregulated				
1.	PGSC0003DMG400034790	Chr_ST4.03ch08:53642228-53644070	P69B protein	−11.003
2.	PGSC0003DMG402024140	Chr_ST4.03ch08:3167462-3169938	PAE	−10.859
3.	PGSC0003DMG400001598	Chr_ST4.03ch01:87145670-87146801	Snakin-2	−10.513
4.	PGSC0003DMG402010991	Chr_ST4.03ch10:54538508-54540606	Cytochrome P450 hydroxylase	−8.777
5.	PGSC0003DMG400029830	Chr_ST4.03ch10:57993306-57994678	Glucan endo-1,3-beta-D-glucosidase	−8.706
6.	PGSC0003DMG400000776	Chr_ST4.03ch04:59015346-59016839	Extensin (ext)	−8.361
7.	PGSC0003DMG400003531	Chr_ST4.03ch02:40090040-40090994	Dhn1 protein	−7.903
8.	PGSC0003DMG400017278	Chr_ST4.03ch07:50526967-50529430	Receptor-like kinase	−7.709
9.	PGSC0003DMG400009530	Chr_ST4.03ch08:2364263-2365922	WRKY transcription factor 3	−6.442
10.	PGSC0003DMG400001204	Chr_ST4.03ch01:46348797-46349646	Ca2+-binding protein 1	−6.217
ii) CPH62				
Upregulated				
1.	PGSC0003DMG400034882	Chr_ST4.03ch07:41317610-41319068	Glucosyltransferase	5.521
2.	PGSC0003DMG402019255	Chr_ST4.03ch07:54348410-54350031	Pectinesterase	5.124
3.	PGSC0003DMG400000493	Chr_ST4.03ch02:47048112-47051618	Carbonic anhydrase	4.996
4.	PGSC0003DMG400022263	Chr_ST4.03ch07:55888668-55891111	Fructose-bisphosphate aldolase	4.720
5.	PGSC0003DMG401021057	Chr_ST4.03ch00:36077457-36079291	Cytochrome P450	3.805
6.	PGSC0003DMG400001599	Chr_ST4.03ch01:87104707-87119208	Pentatricopeptide repeat-containing protein	3.771
7.	PGSC0003DMG400027276	Chr_ST4.03ch04:10137639-10143624	Mg protoporphyrin IX chelatase	3.567
8.	PGSC0003DMG400010223	Chr_ST4.03ch02:32419588-32421499	Phytophthora-inhibited protease 1	3.532
9.	PGSC0003DMG401018223	Chr_ST4.03ch01:79287173-79291540	Ferric-chelate reductase	3.459
10.	PGSC0003DMG400002172	Chr_ST4.03ch09:1002479-1004024	Glutathione S-transferase T2	3.395
Downregulated				
1.	PGSC0003DMG400020017	Chr_ST4.03ch01:45137501-45139268	Lichenase	−6.482
2.	PGSC0003DMG400001948	Chr_ST4.03ch08:46916743-46925962	Copalyl diphosphate synthase	−6.114
3.	PGSC0003DMG400003057	Chr_ST4.03ch08:54294866-54295826	Osmotin	−5.930
4.	PGSC0003DMG400022430	Chr_ST4.03ch02:34338338-34340696	Polyphenoloxidase	−5.708
5.	PGSC0003DMG400031457	Chr_ST4.03ch03:17248039-17249552	Phenylalanine ammonia-lyase 1	−5.487
6.	PGSC0003DMG400029830	Chr_ST4.03ch10:57993306-57994678	Glucan endo-1,3-beta-D-glucosidase	−4.888
7.	PGSC0003DMG400006393	Chr_ST4.03ch04:65678794-65684380	Aux/IAA protein	−4.849
8.	PGSC0003DMG400006814	Chr_ST4.03ch01:66086407-66087912	AN1-like transcription factor	−4.811
9.	PGSC0003DMG400015219	Chr_ST4.03ch03:50225872-50226839	Miraculin	−4.811
10.	PGSC0003DMG400021508	Chr_ST4.03ch04:67179445-67180400	C2H2-type zinc finger protein	−4.778
iii) JAM07				
Upregulated				
1.	PGSC0003DMG400014836	Chr_ST4.03ch08: 18104826-18105445	Steroid binding protein	12.36
2.	PGSC0003DMG400027289	Chr_ST4.03ch04: 10645186-10647010	Glucosyltransferase	12.02
3.	PGSC0003DMG400004008	Chr_ST4.03ch02: 43561825-43570544	PHCLF2	11.75
4.	PGSC0003DMG400027888	Chr_ST4.03ch07: 38959941-38964852	Cysteine proteinase 3	11.57
5.	PGSC0003DMG400028702	Chr_ST4.03ch04: 46543274-46551909	Cycloartenol synthase	11.12
6.	PGSC0003DMG400008794	Chr_ST4.03ch06: 55652701-55654197	Major latex	10.25
7.	PGSC0003DMG400024062	Chr_ST4.03ch03: 2723057-2725494	Expansin	10.05
8.	PGSC0003DMG400046796	Chr_ST4.03ch10: 33569084-33572658	Protein kinase atmrk1	9.97
9.	PGSC0003DMG400027947	Chr_ST4.03ch09: 31126980-31130371	RING finger protein	9.90
10.	PGSC0003DMG400008212	Chr_ST4.03ch08: 34753042-34753447	Abscisic insensitive 1B	9.87
Downregulated				
1.	PGSC0003DMG400015318	Chr_ST4.03ch12: 609681-611652	Metallothionein	−15.31
2.	PGSC0003DMG400034790	Chr_ST4.03ch08: 53642229-53644070	P69B protein	−14.73
3.	PGSC0003DMG400023922	Chr_ST4.03ch09: 7127519-7128055	Cytoplasmic small heat shock protein class I	−14.05
4.	PGSC0003DMG400016623	Chr_ST4.03ch09: 60489530-60491348	Cytochrome P450	−13.94
5.	PGSC0003DMG400015804	Chr_ST4.03ch09: 45371785-45378871	26S proteasome subunit 4	−13.54
6.	PGSC0003DMG400008419	Chr_ST4.03ch11: 17970826-17973912	LRR receptor-like serine/threonine-protein kinase	−13.07
7.	PGSC0003DMG400017127	Chr_ST4.03ch07: 17631058-17636043	Cyclin-dependent protein kinase	−12.88
8.	PGSC0003DMG400024232	Chr_ST4.03ch04: 22066979-22070659	Spermidine synthase 1	−12.75
9.	PGSC0003DMG400002028	Chr_ST4.03ch09: 7119128-7119829	Cytoplasmic small heat shock protein class I	−12.52
10.	PGSC0003DMG400031523	Chr_ST4.03ch09: 60113112-60115182	Glycine-rich cell wall structural protein 1.8	−12.45
iv) MCD24				
Upregulated				
1.	PGSC0003DMG400035689	Chr_ST4.03ch06: 53529488-53530984	Flavonoid glucoyltransferase UGT73N1	12.38
2.	PGSC0003DMG400024690	Chr_ST4.03ch01: 76972977-76976319	Lipoxygenase	10.98
3.	PGSC0003DMG402028957	Chr_ST4.03ch07: 46804099-46805487	Glycine-rich cell wall structural protein 1	10.91
4.	PGSC0003DMG400007701	Chr_ST4.03ch10: 26029370-26033562	Lactose permease	10.77
5.	PGSC0003DMG400027947	Chr_ST4.03ch09: 31126980-31130371	RING finger protein	10.65
6.	PGSC0003DMG400013439	Chr_ST4.03ch03: 343737-347232	Aspartic proteinase oryzasin-1	10.52
7.	PGSC0003DMG400006021	Chr_ST4.03ch11: 8463730-8466858	Amino acid transporter	10.45
8.	PGSC0003DMG400034893	Chr_ST4.03ch01: 27844087-27844772	Transposon protein, CACTA, En/Spm sub-class	10.45
9.	PGSC0003DMG400004008	Chr_ST4.03ch02: 43561825-43570544	PHCLF2	10.39
10.	PGSC0003DMG400003097	Chr_ST4.03ch02: 30665412-30669175	Flavonol synthase/flavanone 3-hydroxylase	10.36
Downregulated				
1.	PGSC0003DMG400020341	Chr_ST4.03ch09: 829873-831558	17.5 kDa class I heat shock protein	−14.20
2.	PGSC0003DMG402021713	Chr_ST4.03ch02: 35575960-35576825	(-)-a-terpineol synthase	−13.09
3.	PGSC0003DMG400023086	Chr_ST4.03ch03: 13287247-13292137	Superoxide dismutase [Cu-Zn]	−12.82
4.	PGSC0003DMG400000978	Chr_ST4.03ch03: 15753246-15758049	Acyl-[acyl-carrier-protein] desaturase, chloroplastic	−12.26
5.	PGSC0003DMG400031360	Chr_ST4.03ch10: 51857269-51859786	UDP-glucoronosyl/UDP-glucosyl transferase family protein	−11.96
6.	PGSC0003DMG400020255	Chr_ST4.03ch02: 47983144-47987190	Mitochondrial import receptor subunit TOM20	−11.93
7.	PGSC0003DMG400029380	Chr_ST4.03ch12: 57950911-57954167	Tryptophan synthase beta chain	−11.93
8.	PGSC0003DMG400033662	Chr_ST4.03ch04: 15290482-15295203	Hydrolase/protein serine/threonine phosphatase	−11.87
9.	PGSC0003DMG400018671	Chr_ST4.03ch03: 3142976-3146980	Heterogeneous nuclear ribonucleoprotein	−11.78
10.	PGSC0003DMG400011235	Chr_ST4.03ch10: 745671-746196	Steroid binding protein	−11.64
v) PLD-47				
Upregulated				
1.	PGSC0003DMG400027888	Chr_ST4.03ch07: 38959941-38964852	Cysteine proteinase 3	12.24
2.	PGSC0003DMG400010129	Chr_ST4.03ch03: 43950496-43951544	Aspartic protease inhibitor 10	12.17
3.	PGSC0003DMG400013830	Chr_ST4.03ch08: 37019719-37023974	Major latex protein	11.65
4.	PGSC0003DMG400006956	Chr_ST4.03ch02: 26445716-26447338	Carbonic anhydrase	11.29
5.	PGSC0003DMG400046796	Chr_ST4.03ch10: 33569084-33572658	Protein kinase atmrk1	11.23
6.	PGSC0003DMG400009267	Chr_ST4.03ch11: 14382374-14383072	Proteinase inhibitor	10.85
7.	PGSC0003DMG400014836	Chr_ST4.03ch08: 18104826-18105445	Steroid binding protein	10.83
8.	PGSC0003DMG400020375	Chr_ST4.03ch12: 12183735-12190583	Endonuclease/exonuclease/phosphatase family protein	10.55
9.	PGSC0003DMG400028702	Chr_ST4.03ch04: 46543274-46551909	Cycloartenol synthase	10.18
10.	PGSC0003DMG400027947	Chr_ST4.03ch09: 31126980-31130371	RING finger protein	10.11
Downregulated				
1.	PGSC0003DMG400006368	Chr_ST4.03ch04: 60659933-60661295	Cytochrome P450 92B1	−14.01
2.	PGSC0003DMG400023922	Chr_ST4.03ch09: 7127519-7128055	Cytoplasmic small heat shock protein class I	−13.66
3.	PGSC0003DMG400016623	Chr_ST4.03ch09: 60489530-60491348	Cytochrome P450	−13.55
4.	PGSC0003DMG401004840	Chr_ST4.03ch06: 48841901-48846236	Phosphatase	−13.33
5.	PGSC0003DMG400014007	Chr_ST4.03ch05: 19928205-19934931	Protein disulfide isomerase	−13.23
6.	PGSC0003DMG400015804	Chr_ST4.03ch09: 45371785-45378871	26S proteasome subunit 4	−13.15
7.	PGSC0003DMG402031759	Chr_ST4.03ch02: 32994672-32997447	Phospholipase A1	−13.07
8.	PGSC0003DMG400030165	Chr_ST4.03ch04: 9478743 - 9479260	Avr9/Cf-9 rapidly elicited protein 180	−12.67
9.	PGSC0003DMG400020377	Chr_ST4.03ch12: 12139743-12145702	70 kDa subunit of replication protein A	−12.67
10.	PGSC0003DMG402021713	Chr_ST4.03ch02: 35575960-35576825	(−)-a-terpineol synthase	−12.43

*DEGs analysis was performed using Kufri Bahar (KB) as control between wild species, *viz.*, PIN45 (*Solanum pinnatisectum*, CGN 17745), CPH62 (*S. cardiophyllum*, PI 283062), JAM07 (*S. jamesii*, PI 498407), MCD24 (*S. microdontum*, PI 218224), and PLD47 (*S. polyadenium*, CGN 17747). Gene expression is expressed in term of log_2_ fold change value.

Some of the selected upregulated genes across all the species were proline-rich protein and MYB transcription factor MYB139 in PIN45, glucosyltransferase and phytophthora-inhibited protease 1 in CPH62, steroid binding protein and RING finger protein in JAM07, flavonoid glucoyltransferase UGT73N1 and glycine-rich cell wall structural protein 1 in MCD24, and cysteine proteinase 3 and carbonic anhydrase in PLD47. Similarly, important downregulated genes in these wild species were snakin-2 and WRKY transcription factor 3 in PNT45, lichenase and AN1-like transcription factor in CPH62, metallothionein and cyclin-dependent protein kinase in JAM07, superoxide dismutase [Cu–Zn] and UDP-glucoronosyl/UDP-glucosyl transferase family protein in MCD24, and cytoplasmic small heat shock protein class I and phosphatase in PLD47 (**Table 2**).

### Gene Ontology and KEGG pathways analysis

**Figure 5:**
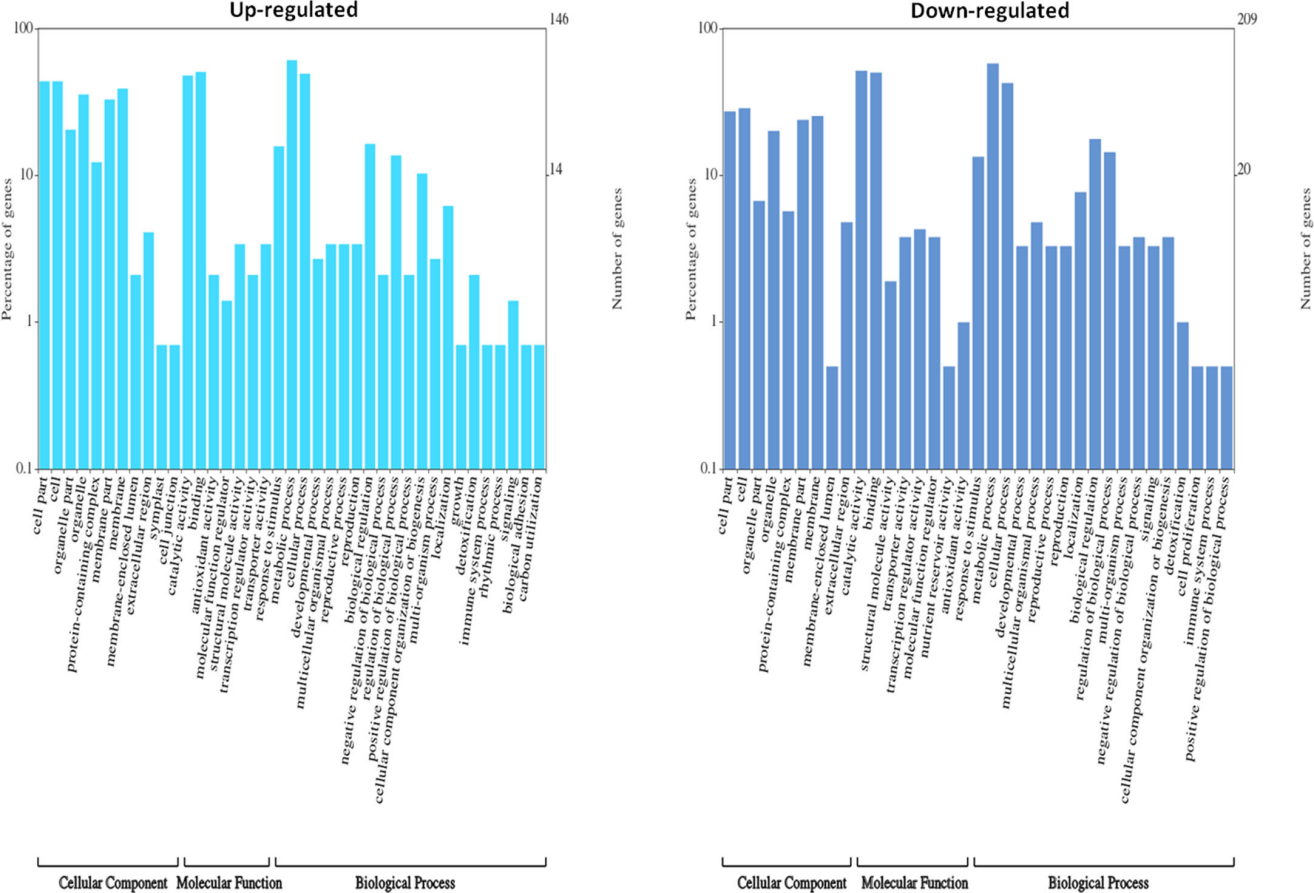
Gene Ontology (GO) characterization for cellular component, molecular function, and biological process of up- and downregulated DEGs in wild potato species PIN45 (*S. pinnatisectum*).

**Figure 6:**
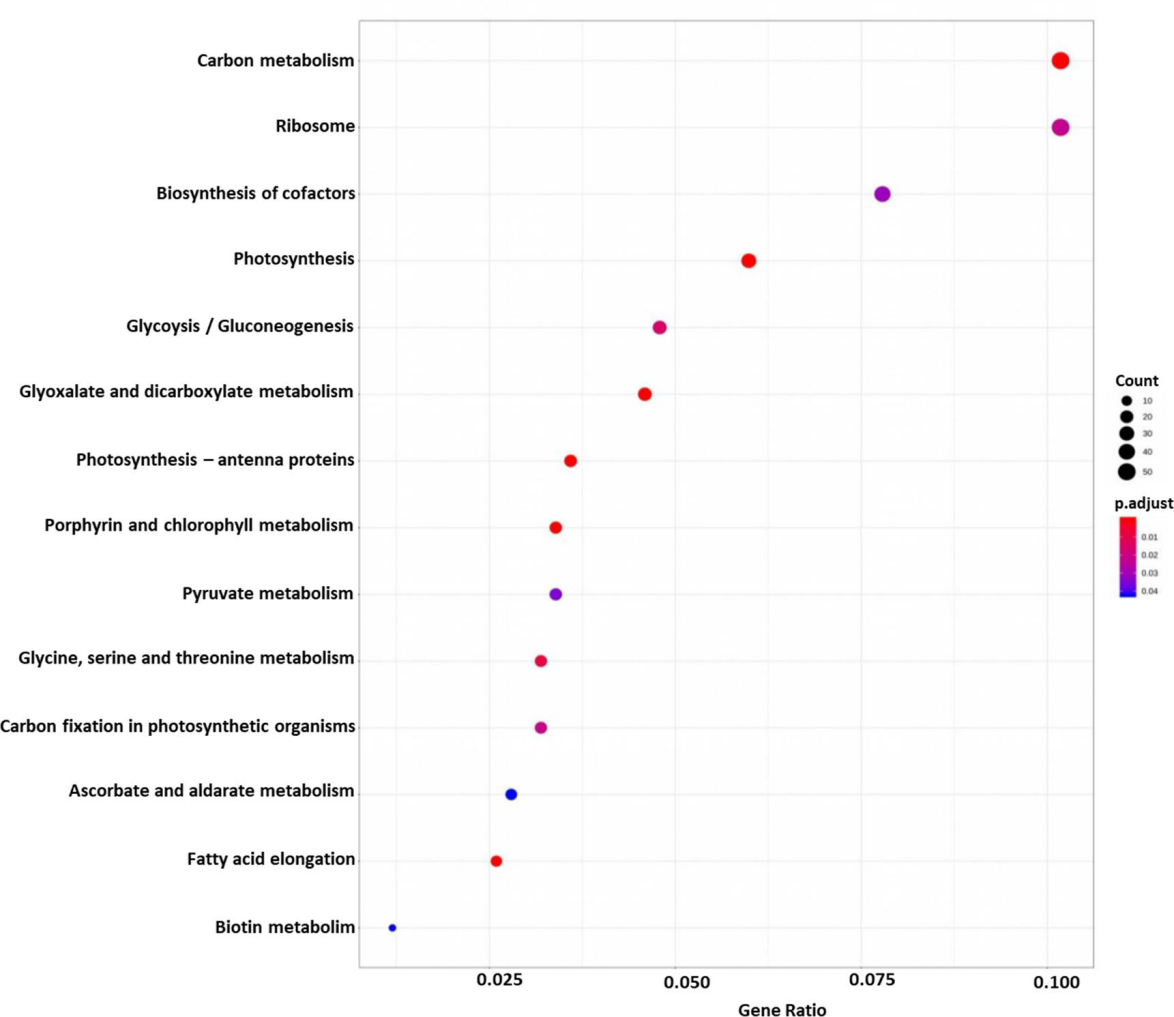
KEGG pathway enrichment analysis of CPH62 for upregulated genes. Red represents more significance; blue represents less significance. Rich factor is the ratio of the differentially expressed gene mapped in the pathway to the total gene number in a certain pathway.

DEGs were functionally characterized with GO terms, namely, molecular function, biological process, and cellular component. Overall, the GO terms predominantly observed in all species were catalytic activity, binding, metabolic process, cellular process, cell, membrane (**Supplementary Datasets 6–10**). Furthermore, DEGs were processed for KEGG pathways and classified into 24 KEGG functional pathways categories, which included KEGG annotated gene counts. Maximum KEGG annotated gene counts were found for signal transduction than for other pathways like translation, carbohydrate metabolism, folding sorting and degradation, amino acid metabolism, energy metabolism, lipid metabolism and transport, catabolism, cell growth and death, and environmental adaptation (**Supplementary Datasets 11–15**). The WEGO plots of PIN45 are depicted for both up- and downregulated genes in **Figure 5**. The rest of the WEGO plots are mentioned in **Supplementary Files** (**Supplementary Figures S4, S5**). KEGG pathway enrichment analysis for CPH62 upregulated genes is depicted in **Figure 6**. In addition, plant–pathogen interaction KEGG mapping information is given in **Supplementary Files** (**Supplementary Figures S6, S7**).

### Real-time quantitative PCR analysis

The selected 10 genes (two genes from each of both up- and downregulated) were validated by RT-qPCR analysis using Kufri Bahar as control like RNA-seq data. RT-qPCR results are consistent with the RNA-seq results with some variation in gene expression values. RT-PCR analysis was carried out in all species such as PNT45 for proline-rich protein and WRKY transcription factor 3, CPH62 for glucosyltransferase and phenylalanine ammonia-lyase 1, JAM07 for steroid-binding protein and LRR receptor-like serine/threonine-protein kinase, MCD24 for glycine-rich cell wall structural protein 1 and 17.5 kDa class I heat shock protein, and PLD-47 for cysteine proteinase 3 and phosphatase (**Supplementary Table S1**).

## Discussion

Wild *Solanum* species are the reservoir of late blight resistance genes in potato. However, the genomic information about wild potato species is limited. In this study, of the 40 genotypes screened, 23 were HR, of which five species and control (Kufri Bahar) were transcriptome analyzed and several DEGs were identified. Our results are in accordance with earlier findings on late blight resistance in potato (Sundaresha et al., 2014; Tiwari et al., 2015). RNA-seq analyses have been conducted earlier by many researchers and discovered multiple genes (e.g., *R* genes, disease resistance, phytohomones, and stress) associated with late blight resistance in potato (Frades et al., 2015; Yang et al., 2018; Yang et al., 2020). Our study also sheds light on improving the understanding of post-inoculation-induced genes associated with late blight resistance in wild potato species, which would serve a vital resource for future studies.

Disease resistance genes play key roles in conferring late blight resistance against *P. infestans* via *R* gene–avirulence *Avr* protein interaction. In this study, we identified a number of induced or suppressed DEGs upon pathogen infection (96-h post-inoculation) in wild species. In these genes, some selected induced genes such as late blight resistance genes (*Rpi-blb2*, PSH-RGH7, TIR-NBS-LRR, R1B-16, and BS2) or disease resistance genes (RGA2 and RGA3), and suppressed genes like disease resistance genes were like CC-NBS-LRR, *R3a*, *Rpi-pta1*, ABC transporter family, Sn-1, and RGA4 in different species. These genes would be very useful for future exploitation of wild species, as they are not crossable with common potato due to the differences in ploidy number and endosperm balance number (Jansky, 2006). Our results are in agreement with earlier finding using microarray technology, indicating a strong association of disease resistance genes in conferring late blight resistance in potato cv. Kufri Girdhai (Sundaresha et al., 2014; Singh et al., 2016). Allele mining study elucidates the involvement of the *R* genes (*Rpi-blb1*) in wild species (Tiwari et al., 2015). Another study advocates that wild species (*S. pinnatisectum*)-originated genes confer high resistance in interspecific potato somatic hybrids derived through protoplast fusion (Sarkar et al., 2011; Tiwari et al., 2021). Furthermore, a transcriptomics study identifies multiple upregulated NBS-LRR genes suppressing *P. infestans* infection and providing resistance against late blight in *S. pinnatisectum* (Gu et al., 2020). Indeed, an array of defense response is activated in the plant cells upon pathogen infection, and differential response of genes is noticed (Sarowar et al., 2011). Largely, R genes containing a central nucleotide binding and hydrolyzing domain (NB-ARC) and a C-terminal leucine-rich repeat (LRR) domain play a vital role in conferring disease resistance (Bozkurt et al., 2011). Additionally, phytohormones like salicylic acid, jasmonic acid, abscisic acid, ethylene, and brassinosteroids-mediated defense signaling pathways play key roles in providing late blight resistance in potato (Duan et al., 2020). Recently, it has been demonstrated that exogenous ethylene application induces ethylene signaling pathways in defense response against late blight in potato SD20 (Yang et al., 2020). Furthermore, they suggest that multiple signaling pathways are involved in defense response involving ethylene, salicylic acid, jasmonic acid, abscisic acid, auxin, cytokinin, and gibberellins in SD20. Frades et al. (2015) developed a novel workflow correlating RNA-seq data to *P. infestans* resistance levels in wild *Solanum* species and potato clones and identified 400 expressed putative *R* genes in resistant clones (Sarpo Mira and SW93-1015) than susceptible cv. Desiree. Thus, we enrich knowledge and genomics information particularly on gene abundance in wild potato species for future studies.

Transcription factors (TFs) play a very vital role in providing late blight resistance in potato. TFs are involved in genes expression and metabolic pathways in disease response. Here, we identified a number of TFs (upregulated/downregulated) in wild species in response to pathogen attack to provide host resistance. A few important upregulated TFs were GRAS, MADS, MYB, and AP2, whereas downregulated TFs were AP2/ERF, BZIP, C2H2L, NAC, BHLH, RWP-RK, WRKY, bHLH62, etc. Our findings are in congruence with previous work on identification of TFs in late blight resistance in potato (Yang et al., 2020). Researchers have identified TFs like heat shock protein, zinc finger ring-box protein-like, NAC22, MYB44, WRKY, and MYB in response to late blight resistance in potato (Kikuchi et al., 2000; Jiang et al., 2017). The importance of zinc finger protein has been evidenced in defense response to late blight resistance in potato (Xiang and Judelson, 2014). A previous study has confirmed the induced expression of the responsive genes in the late blight resistance signaling pathway, such as WRKY, ERF, MAPK, and NBS-LRR family genes (Xiang and Judelson, 2014). Frades et al. (2015) identified zinc knuckle family proteins in resistant cultivar, whereas transmembrane transport and protein acylation were observed in susceptible genotypes through transcriptome profiling in potato. Our findings strengthen the knowledge on the availability of TFs governing genes expression for phenotypic response in plants against late blight disease in wild potato species.

A number of stress-responsive genes, phytohormones, photosynthesis, and starch metabolism genes are known in defense response against *P. infestans* in potato. In this study, a few DEGs involved in starch metabolism were glucosyltransferase and UDP-glucose:glucosyltransferase, whereas stress-responsive genes were abscisic acid receptor PYL4, proline-rich protein, serine–threonine protein kinase, and leucine-rich repeat resistance protein, etc. Our findings are supported by many previous results on stress- and phytohormones-related genes imparting late blight resistance in potato. Gao and Bradeen (2016) suggested that *R* gene and ethylene and stress-responsive genes contribute to the *RB* gene-mediated incompatible potato–*P. infestans* interactions in both the foliage and tubers of potato. Downregulation of photosynthesis and upregulation of ethylene and stress-related genes play a key role in conferring late blight resistance in potato. Methyltransferase genes are also induced in late blight-resistant wild species. The caffeoyl-CoA O-methyltransferase gene mutant enhances late blight resistance through cell wall reinforcement by genome editing in potato cv. Russet Burbank (Hegde et al., 2021). Recently, a comparative transcriptome analysis of the *R3a* and *Avr3a* genes-mediated defense response in transgenic tomato revealed that phenylpropanoid biosynthesis pathway, plant–pathogen interaction, and plant hormone signal transduction pathways are significantly upregulated, whereas carbon metabolism and photosynthesis pathways genes were downregulated upon pathogen infection (Xue et al., 2021). This also includes TFs such as WRKY, MYB, and NAC associated with disease resistance and endogenous phytohormones such as salicylic acid and ethylene pathways, and pathogenesis-related genes were significantly induced. Cytochrome P450 plays a key role in enhancing plant resistance via their involvement in the jasmonic acid and ethylene signaling pathways in potato (Duan et al., 2020). The study indicates the role of protein kinases, mainly calcium-dependent protein kinases (CDPKs) and leucine-rich repeat receptor-like protein kinases (LRR-RKs), in pathogen resistance in lentil (Khorramdelazad et al., 2018). The role of photosynthesis is well-described in plant growth and development. Burra et al. (2018) suggest that most of the genes associated with photosynthesis pathways are downregulated upon *P. infestans* inoculation, leading to hypersensitive response and leaf lesion. Overall, we identified a number of key genes imparting late blight resistance in wild potato species.

## Conclusions

Of total 40 potato genotypes, we investigated five highly resistant wild species, *viz*., PIN45 (*S. pinnatisectum*), CPH62 (*S. cardiophyllum*), JAM07 (*S. jamesii*), MCD24 (*S. microdontum*), and PLD47 (*S. polyadenium*). We provide a number of key genes associated with late blight resistance and multiple signaling pathways such as disease resistance protein, TFs, stress-responsive genes, starch metabolism genes, phytohormones, and photosynthesis-related genes. In addition, there were numerous genes involved in imparting late blight resistance in wild species. These wild species are potential source of potato improvement, applying conventional or molecular approaches. Future research is required on the functional characterization of candidate genes via transgenic or genome editing.

## Data availability statement

The RNA-sequence data have been deposited with the NCBI database Bioproject ID: PRJNA744887 and PRJNA836253.

## Author contributions

JT designed the study. NB, JT, RZ, and TB performed research work. SS, DD, and VK performed late blight test. JT wrote the original draft of the manuscript. RS, AT, and CK critically edited the manuscript. All authors read and confirmed the manuscript for publication.
