# Supplementary material for: Screening of wild species and transcriptome profiling to identify differentially regulated genes in response to late blight resistance in potato
Source: Front Plant Sci. 2023 Jul 12;14:1212135. doi: 10.3389/fpls.2023.1212135 (PMC10368984; doi:10.3389/fpls.2023.1212135)
Supplement: Supplementary file 1 [file DataSheet_1.zip › Suppl. files_R1_10-6-23/Suppl. Figs. Heat maps.pptx]

## Slide 1
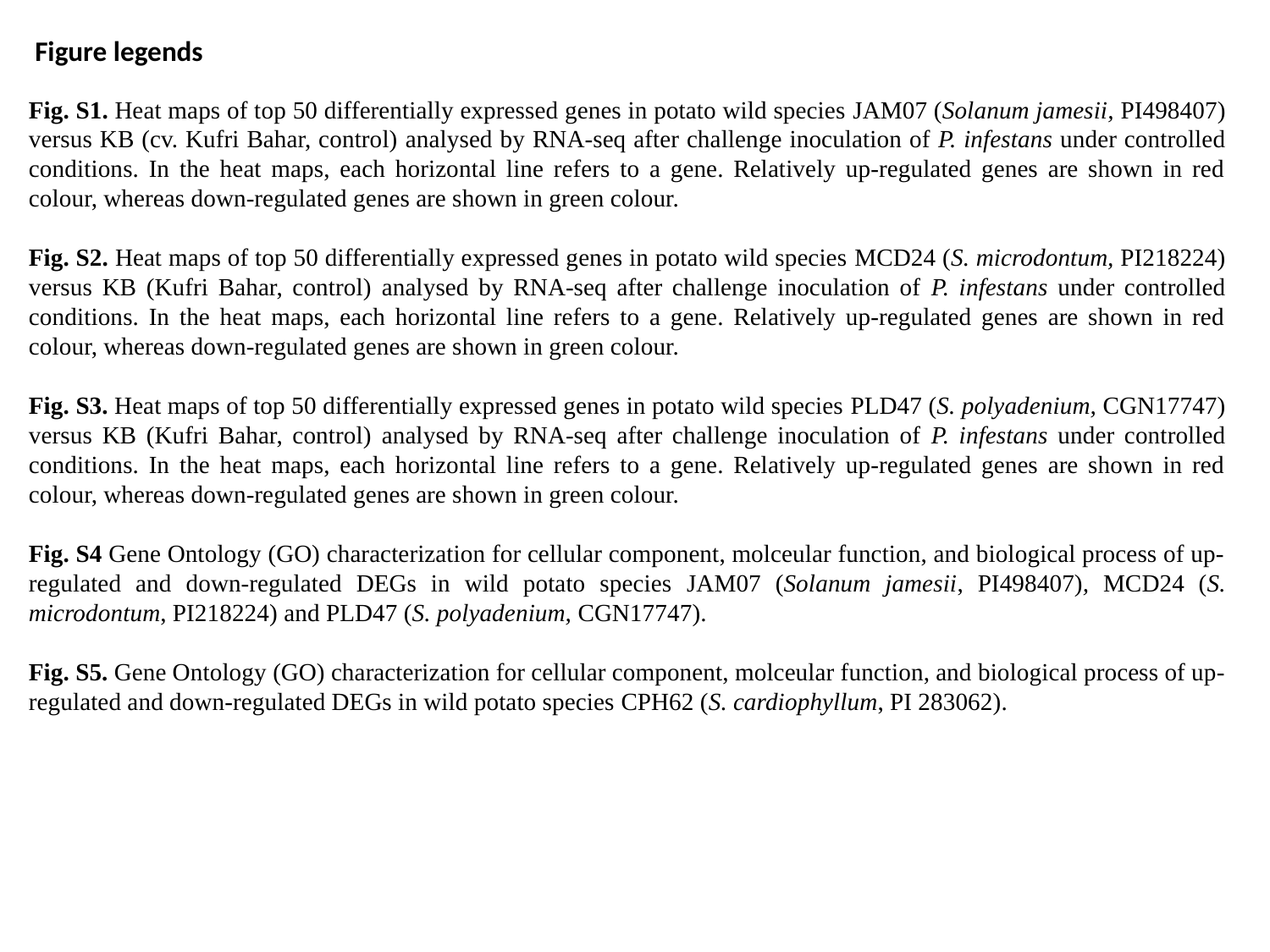

Figure legends
Fig. S1. Heat maps of top 50 differentially expressed genes in potato wild species JAM07 (Solanum jamesii, PI498407) versus KB (cv. Kufri Bahar, control) analysed by RNA-seq after challenge inoculation of P. infestans under controlled conditions. In the heat maps, each horizontal line refers to a gene. Relatively up-regulated genes are shown in red colour, whereas down-regulated genes are shown in green colour.
Fig. S2. Heat maps of top 50 differentially expressed genes in potato wild species MCD24 (S. microdontum, PI218224) versus KB (Kufri Bahar, control) analysed by RNA-seq after challenge inoculation of P. infestans under controlled conditions. In the heat maps, each horizontal line refers to a gene. Relatively up-regulated genes are shown in red colour, whereas down-regulated genes are shown in green colour.
Fig. S3. Heat maps of top 50 differentially expressed genes in potato wild species PLD47 (S. polyadenium, CGN17747) versus KB (Kufri Bahar, control) analysed by RNA-seq after challenge inoculation of P. infestans under controlled conditions. In the heat maps, each horizontal line refers to a gene. Relatively up-regulated genes are shown in red colour, whereas down-regulated genes are shown in green colour.
Fig. S4 Gene Ontology (GO) characterization for cellular component, molceular function, and biological process of up-regulated and down-regulated DEGs in wild potato species JAM07 (Solanum jamesii, PI498407), MCD24 (S. microdontum, PI218224) and PLD47 (S. polyadenium, CGN17747).
Fig. S5. Gene Ontology (GO) characterization for cellular component, molceular function, and biological process of up-regulated and down-regulated DEGs in wild potato species CPH62 (S. cardiophyllum, PI 283062).

## Slide 2
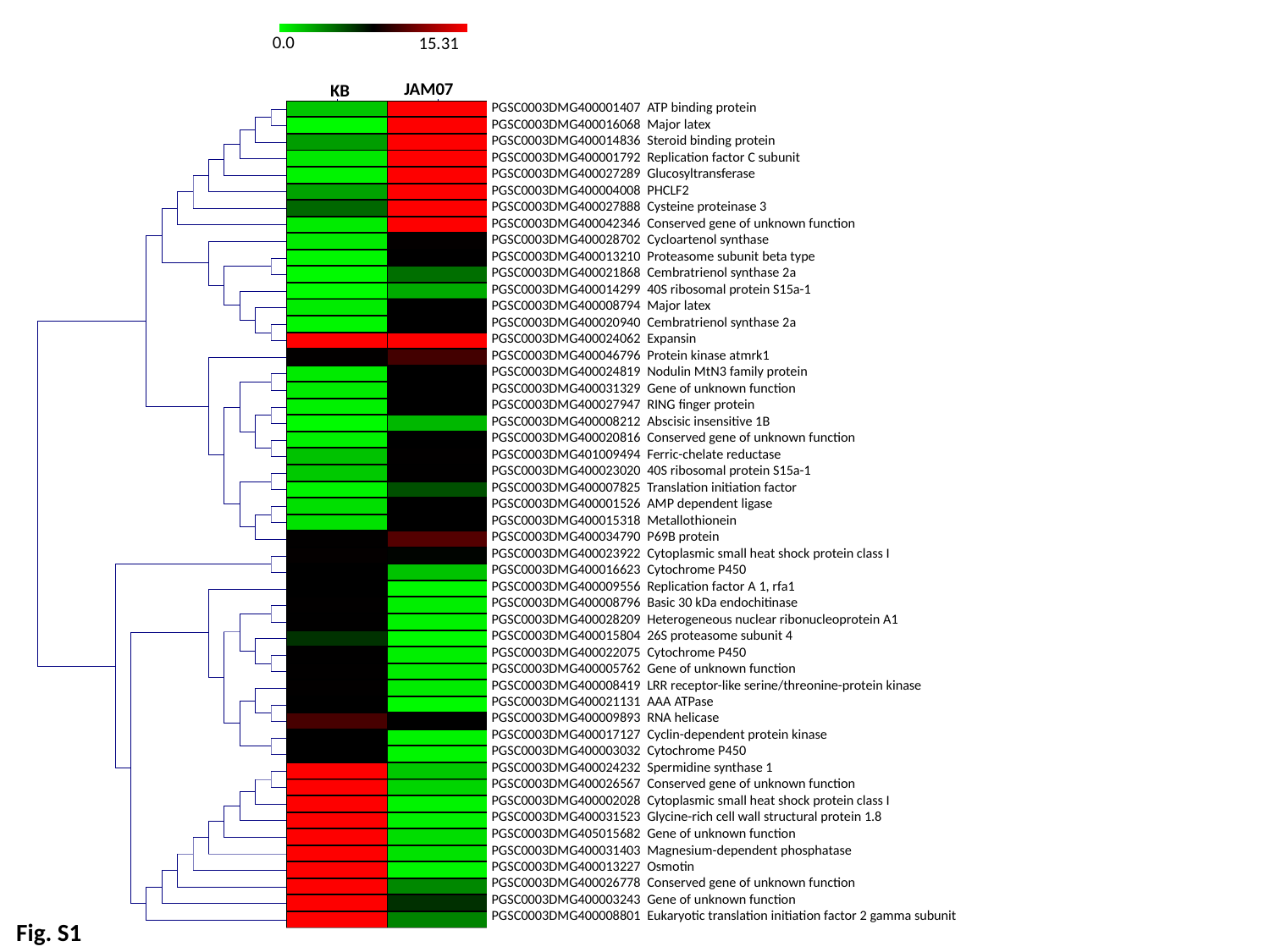

0.0
15.31
JAM07
KB
PGSC0003DMG400001407 ATP binding protein
PGSC0003DMG400016068 Major latex
PGSC0003DMG400014836 Steroid binding protein
PGSC0003DMG400001792 Replication factor C subunit
PGSC0003DMG400027289 Glucosyltransferase
PGSC0003DMG400004008 PHCLF2
PGSC0003DMG400027888 Cysteine proteinase 3
PGSC0003DMG400042346 Conserved gene of unknown function
PGSC0003DMG400028702 Cycloartenol synthase
PGSC0003DMG400013210 Proteasome subunit beta type
PGSC0003DMG400021868 Cembratrienol synthase 2a
PGSC0003DMG400014299 40S ribosomal protein S15a-1
PGSC0003DMG400008794 Major latex
PGSC0003DMG400020940 Cembratrienol synthase 2a
PGSC0003DMG400024062 Expansin
PGSC0003DMG400046796 Protein kinase atmrk1
PGSC0003DMG400024819 Nodulin MtN3 family protein
PGSC0003DMG400031329 Gene of unknown function
PGSC0003DMG400027947 RING finger protein
PGSC0003DMG400008212 Abscisic insensitive 1B
PGSC0003DMG400020816 Conserved gene of unknown function
PGSC0003DMG401009494 Ferric-chelate reductase
PGSC0003DMG400023020 40S ribosomal protein S15a-1
PGSC0003DMG400007825 Translation initiation factor
PGSC0003DMG400001526 AMP dependent ligase
PGSC0003DMG400015318 Metallothionein
PGSC0003DMG400034790 P69B protein
PGSC0003DMG400023922 Cytoplasmic small heat shock protein class I
PGSC0003DMG400016623 Cytochrome P450
PGSC0003DMG400009556 Replication factor A 1, rfa1
PGSC0003DMG400008796 Basic 30 kDa endochitinase
PGSC0003DMG400028209 Heterogeneous nuclear ribonucleoprotein A1
PGSC0003DMG400015804 26S proteasome subunit 4
PGSC0003DMG400022075 Cytochrome P450
PGSC0003DMG400005762 Gene of unknown function
PGSC0003DMG400008419 LRR receptor-like serine/threonine-protein kinase
PGSC0003DMG400021131 AAA ATPase
PGSC0003DMG400009893 RNA helicase
PGSC0003DMG400017127 Cyclin-dependent protein kinase
PGSC0003DMG400003032 Cytochrome P450
PGSC0003DMG400024232 Spermidine synthase 1
PGSC0003DMG400026567 Conserved gene of unknown function
PGSC0003DMG400002028 Cytoplasmic small heat shock protein class I
PGSC0003DMG400031523 Glycine-rich cell wall structural protein 1.8
PGSC0003DMG405015682 Gene of unknown function
PGSC0003DMG400031403 Magnesium-dependent phosphatase
PGSC0003DMG400013227 Osmotin
PGSC0003DMG400026778 Conserved gene of unknown function
PGSC0003DMG400003243 Gene of unknown function
PGSC0003DMG400008801 Eukaryotic translation initiation factor 2 gamma subunit
Fig. S1

## Slide 3
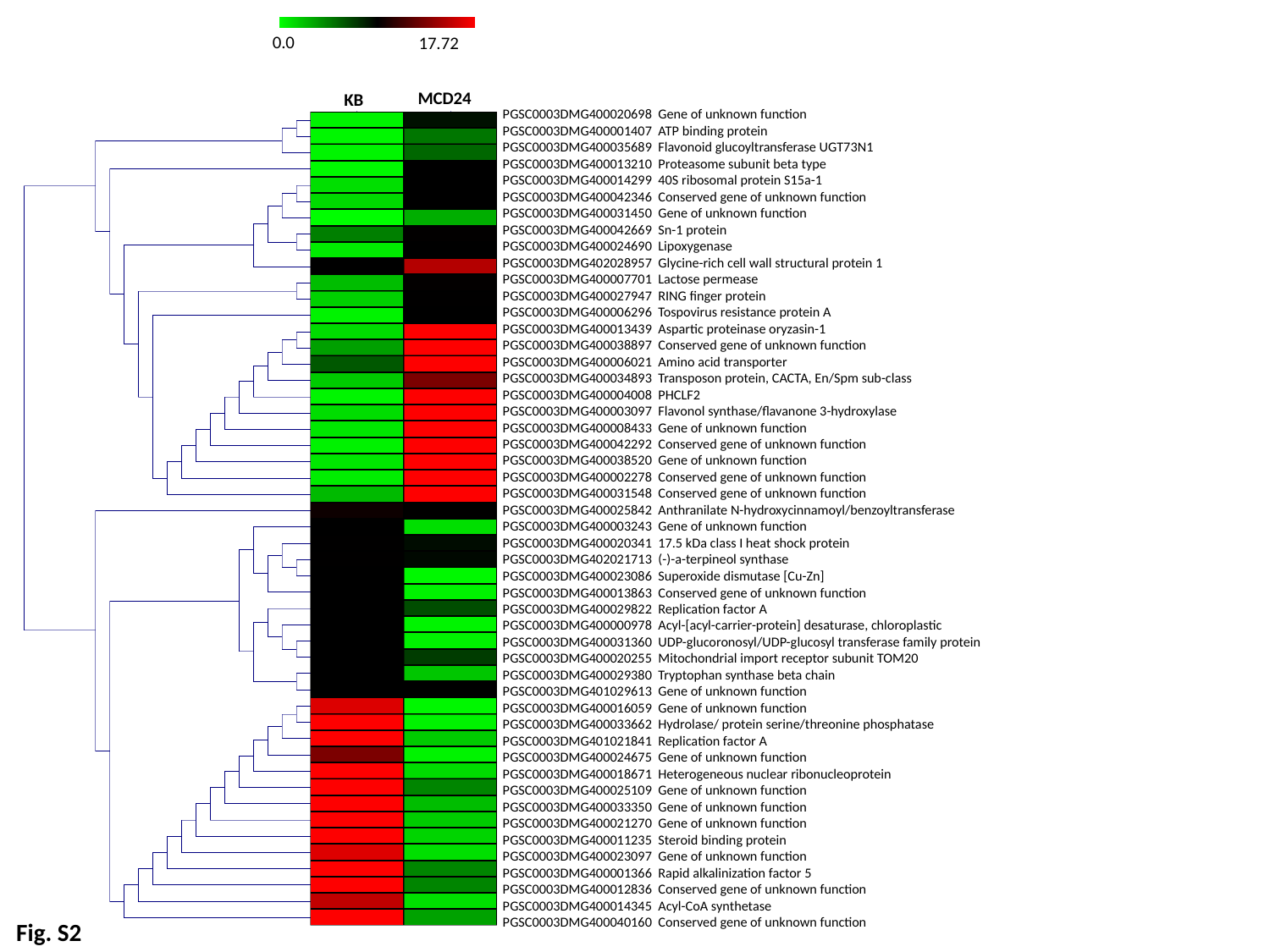

0.0
17.72
MCD24
KB
PGSC0003DMG400020698 Gene of unknown function
PGSC0003DMG400001407 ATP binding protein
PGSC0003DMG400035689 Flavonoid glucoyltransferase UGT73N1
PGSC0003DMG400013210 Proteasome subunit beta type
PGSC0003DMG400014299 40S ribosomal protein S15a-1
PGSC0003DMG400042346 Conserved gene of unknown function
PGSC0003DMG400031450 Gene of unknown function
PGSC0003DMG400042669 Sn-1 protein
PGSC0003DMG400024690 Lipoxygenase
PGSC0003DMG402028957 Glycine-rich cell wall structural protein 1
PGSC0003DMG400007701 Lactose permease
PGSC0003DMG400027947 RING finger protein
PGSC0003DMG400006296 Tospovirus resistance protein A
PGSC0003DMG400013439 Aspartic proteinase oryzasin-1
PGSC0003DMG400038897 Conserved gene of unknown function
PGSC0003DMG400006021 Amino acid transporter
PGSC0003DMG400034893 Transposon protein, CACTA, En/Spm sub-class
PGSC0003DMG400004008 PHCLF2
PGSC0003DMG400003097 Flavonol synthase/flavanone 3-hydroxylase
PGSC0003DMG400008433 Gene of unknown function
PGSC0003DMG400042292 Conserved gene of unknown function
PGSC0003DMG400038520 Gene of unknown function
PGSC0003DMG400002278 Conserved gene of unknown function
PGSC0003DMG400031548 Conserved gene of unknown function
PGSC0003DMG400025842 Anthranilate N-hydroxycinnamoyl/benzoyltransferase
PGSC0003DMG400003243 Gene of unknown function
PGSC0003DMG400020341 17.5 kDa class I heat shock protein
PGSC0003DMG402021713 (-)-a-terpineol synthase
PGSC0003DMG400023086 Superoxide dismutase [Cu-Zn]
PGSC0003DMG400013863 Conserved gene of unknown function
PGSC0003DMG400029822 Replication factor A
PGSC0003DMG400000978 Acyl-[acyl-carrier-protein] desaturase, chloroplastic
PGSC0003DMG400031360 UDP-glucoronosyl/UDP-glucosyl transferase family protein
PGSC0003DMG400020255 Mitochondrial import receptor subunit TOM20
PGSC0003DMG400029380 Tryptophan synthase beta chain
PGSC0003DMG401029613 Gene of unknown function
PGSC0003DMG400016059 Gene of unknown function
PGSC0003DMG400033662 Hydrolase/ protein serine/threonine phosphatase
PGSC0003DMG401021841 Replication factor A
PGSC0003DMG400024675 Gene of unknown function
PGSC0003DMG400018671 Heterogeneous nuclear ribonucleoprotein
PGSC0003DMG400025109 Gene of unknown function
PGSC0003DMG400033350 Gene of unknown function
PGSC0003DMG400021270 Gene of unknown function
PGSC0003DMG400011235 Steroid binding protein
PGSC0003DMG400023097 Gene of unknown function
PGSC0003DMG400001366 Rapid alkalinization factor 5
PGSC0003DMG400012836 Conserved gene of unknown function
PGSC0003DMG400014345 Acyl-CoA synthetase
PGSC0003DMG400040160 Conserved gene of unknown function
Fig. S2

## Slide 4
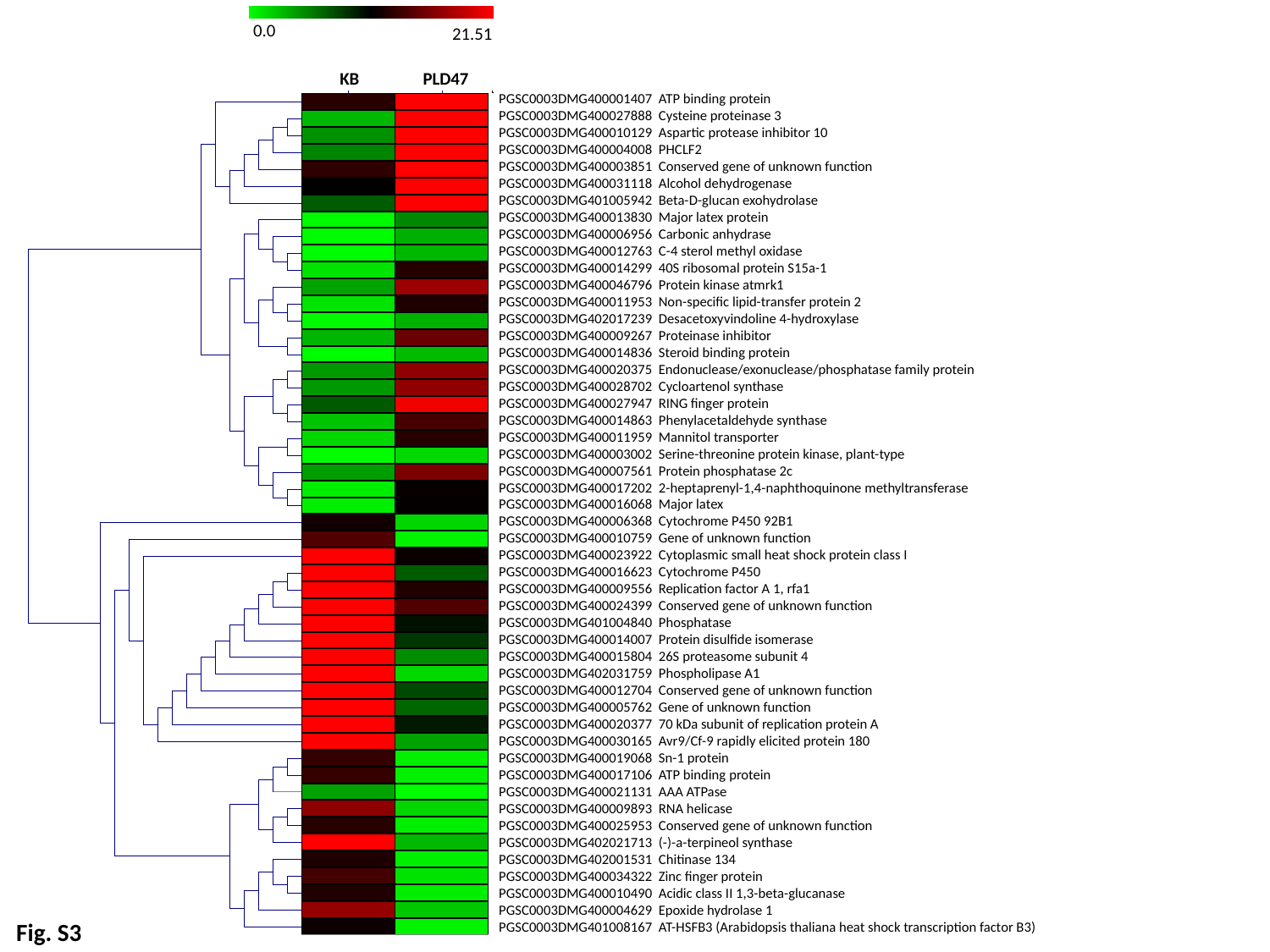

0.0
21.51
KB
PLD47
PGSC0003DMG400001407 ATP binding protein
PGSC0003DMG400027888 Cysteine proteinase 3
PGSC0003DMG400010129 Aspartic protease inhibitor 10
PGSC0003DMG400004008 PHCLF2
PGSC0003DMG400003851 Conserved gene of unknown function
PGSC0003DMG400031118 Alcohol dehydrogenase
PGSC0003DMG401005942 Beta-D-glucan exohydrolase
PGSC0003DMG400013830 Major latex protein
PGSC0003DMG400006956 Carbonic anhydrase
PGSC0003DMG400012763 C-4 sterol methyl oxidase
PGSC0003DMG400014299 40S ribosomal protein S15a-1
PGSC0003DMG400046796 Protein kinase atmrk1
PGSC0003DMG400011953 Non-specific lipid-transfer protein 2
PGSC0003DMG402017239 Desacetoxyvindoline 4-hydroxylase
PGSC0003DMG400009267 Proteinase inhibitor
PGSC0003DMG400014836 Steroid binding protein
PGSC0003DMG400020375 Endonuclease/exonuclease/phosphatase family protein
PGSC0003DMG400028702 Cycloartenol synthase
PGSC0003DMG400027947 RING finger protein
PGSC0003DMG400014863 Phenylacetaldehyde synthase
PGSC0003DMG400011959 Mannitol transporter
PGSC0003DMG400003002 Serine-threonine protein kinase, plant-type
PGSC0003DMG400007561 Protein phosphatase 2c
PGSC0003DMG400017202 2-heptaprenyl-1,4-naphthoquinone methyltransferase
PGSC0003DMG400016068 Major latex
PGSC0003DMG400006368 Cytochrome P450 92B1
PGSC0003DMG400010759 Gene of unknown function
PGSC0003DMG400023922 Cytoplasmic small heat shock protein class I
PGSC0003DMG400016623 Cytochrome P450
PGSC0003DMG400009556 Replication factor A 1, rfa1
PGSC0003DMG400024399 Conserved gene of unknown function
PGSC0003DMG401004840 Phosphatase
PGSC0003DMG400014007 Protein disulfide isomerase
PGSC0003DMG400015804 26S proteasome subunit 4
PGSC0003DMG402031759 Phospholipase A1
PGSC0003DMG400012704 Conserved gene of unknown function
PGSC0003DMG400005762 Gene of unknown function
PGSC0003DMG400020377 70 kDa subunit of replication protein A
PGSC0003DMG400030165 Avr9/Cf-9 rapidly elicited protein 180
PGSC0003DMG400019068 Sn-1 protein
PGSC0003DMG400017106 ATP binding protein
PGSC0003DMG400021131 AAA ATPase
PGSC0003DMG400009893 RNA helicase
PGSC0003DMG400025953 Conserved gene of unknown function
PGSC0003DMG402021713 (-)-a-terpineol synthase
PGSC0003DMG402001531 Chitinase 134
PGSC0003DMG400034322 Zinc finger protein
PGSC0003DMG400010490 Acidic class II 1,3-beta-glucanase
PGSC0003DMG400004629 Epoxide hydrolase 1
PGSC0003DMG401008167 AT-HSFB3 (Arabidopsis thaliana heat shock transcription factor B3)
Fig. S3

## Slide 5
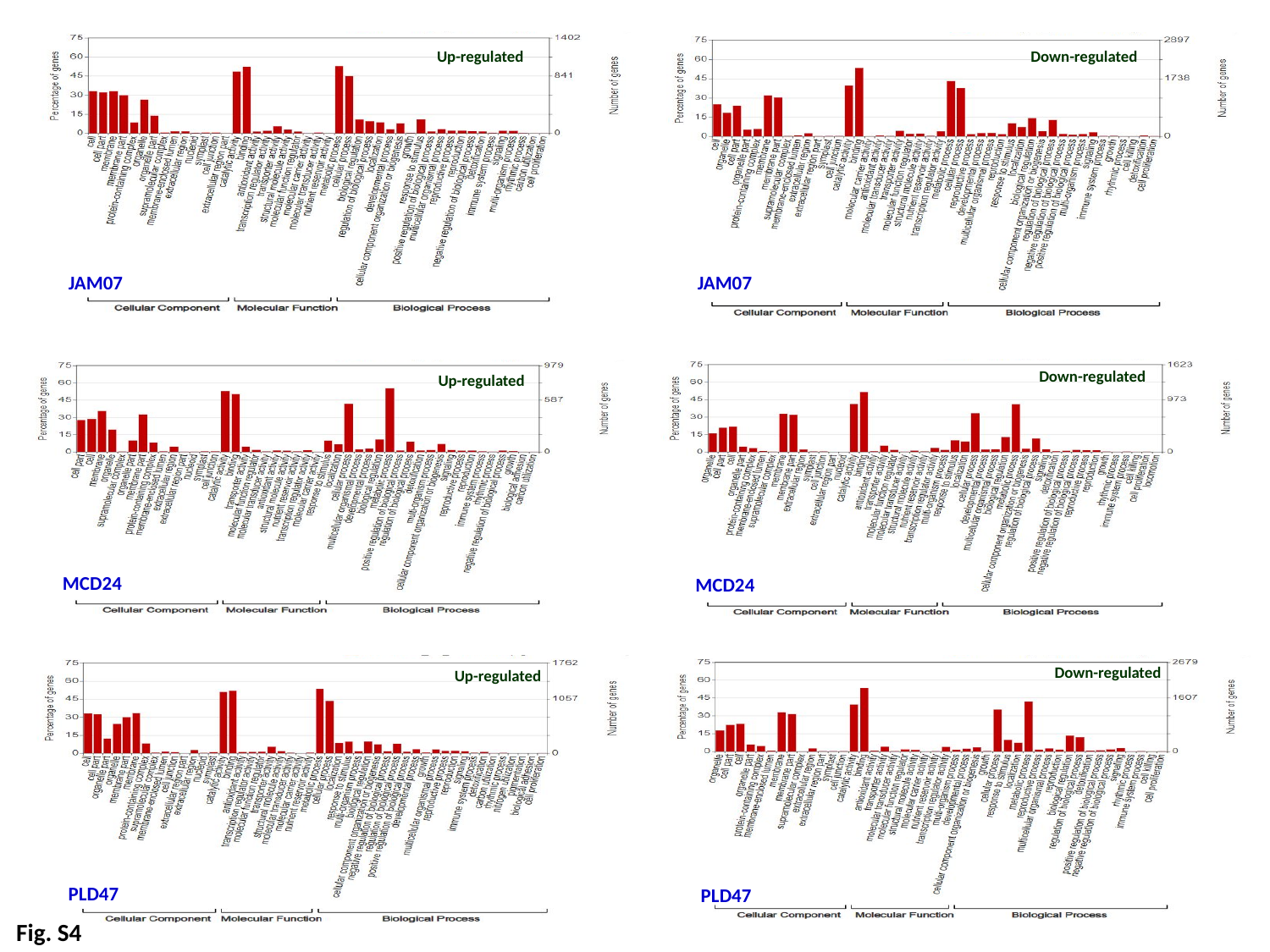

Up-regulated
Down-regulated
JAM07
JAM07
Down-regulated
Up-regulated
MCD24
MCD24
Down-regulated
Up-regulated
PLD47
PLD47
Fig. S4

## Slide 6
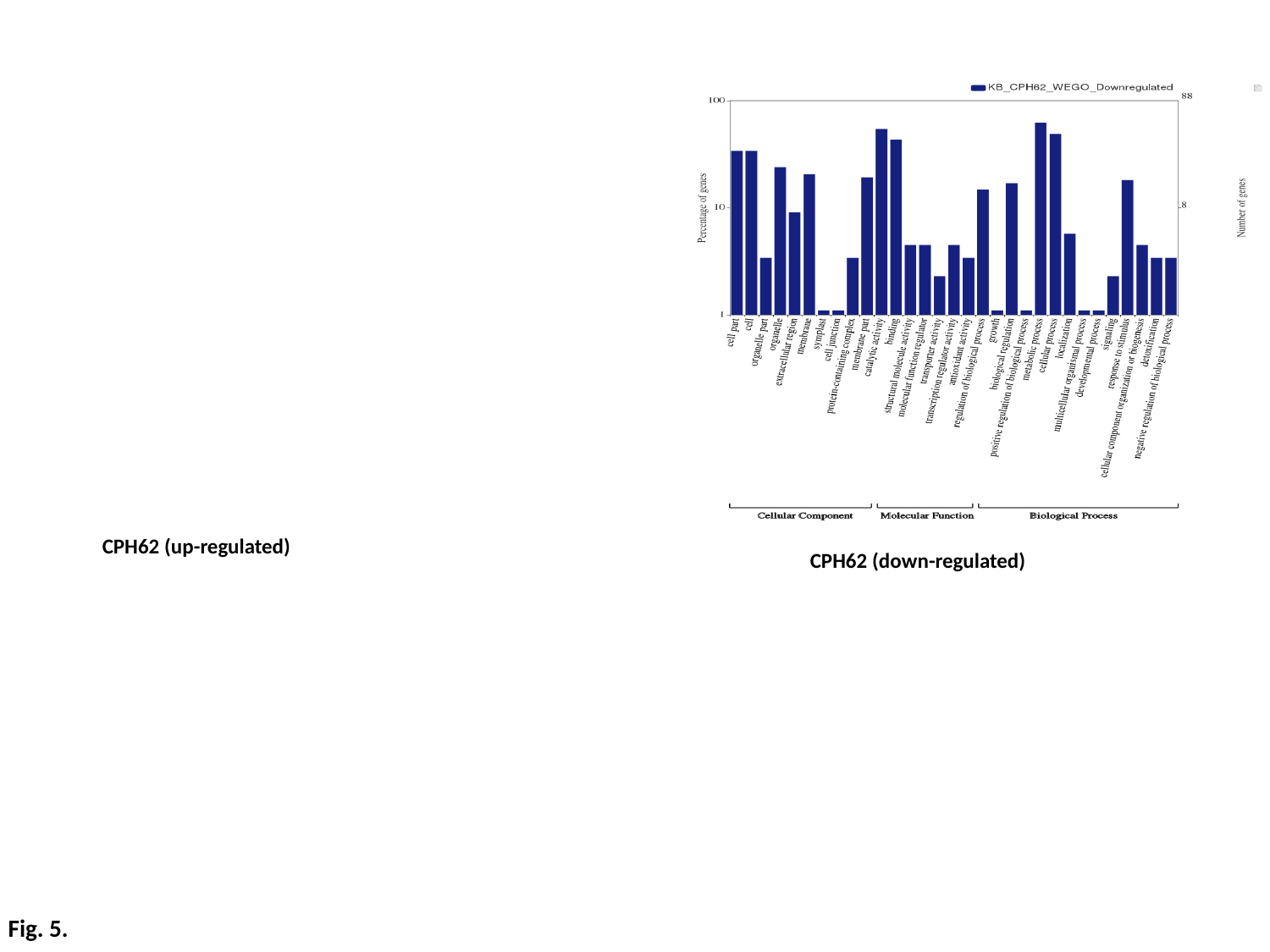

CPH62 (up-regulated)
CPH62 (down-regulated)
Fig. 5.
