# Supplementary material for: Screening of wild species and transcriptome profiling to identify differentially regulated genes in response to late blight resistance in potato
Source: Front Plant Sci. 2023 Jul 12;14:1212135. doi: 10.3389/fpls.2023.1212135 (PMC10368984; doi:10.3389/fpls.2023.1212135)
Supplement: Supplementary file 1 [file DataSheet_1.zip › Suppl. files_R1_10-6-23/Suppl. Table S1_21-4-23.docx]

**Suppl. Table S1.** RT-qPCR analysis of selected genes

| Sr. No | Gene ID | Gene description | Primer sequence (5’→3’) | Gene expression  (Log_2_ FC) | |
| --- | --- | --- | --- | --- | --- |
|  |  |  |  | RNA-seq | RT-qPCR |
| PNT45 | |  |  |  |  |
| 1. | PGSC0003DMG400002880 | Proline-rich protein | F: CTTCCCTCACTCCCATTCTTG  R: GATAGGTGGCAAATGGATAGGG | 5.58 | 4.01 |
| 2. | PGSC0003DMG400009530 | WRKY transcription factor 3 | F: AGAGTTCCGGCAATCAGTTC  R: CCTTTGATCGGCTTTTGACC | -6.44 | -4.27 |
| CPH62 | |  |  |  |  |
| 3. | PGSC0003DMG400034882 | Glucosyltransferase | F: CTGTAGTGTTCTTGTGCTTTGG  R: GCATCTCTATTTTCCCCTTTGG | 5.52 | 3.86 |
| 4. | PGSC0003DMG400031457 | Phenylalanine ammonia-lyase 1 | F: CGAGCAACACAACCAAGATG  R: CCTCCAAATGCCTCAAATCG | -5.48 | -3.72 |
| JAM07 | |  |  |  |  |
| 5. | PGSC0003DMG400014836 | Steroid binding protein | F: GGTAATTCGTTCTATGGTCCCG  R: CTTCGTTCTTGCTCATCTTTGC | 12.36 | 7.52 |
| 6. | PGSC0003DMG400008419 | LRR receptor-like serine/ threonine-protein kinase | F: GACCGCTTGACCAATTTGATG  R: GAATCCAACGACACCAACTG | -13.07 | -8.10 |
| MCD24 | |  |  |  |  |
| 7. | PGSC0003DMG402028957 | Glycine-rich cell wall structural protein 1 | F: CTTGGAATTGAGTGCCATCAC  R: TCTACCAAATCGTCCACAACC | 10.91 | 6.24 |
| 8. | PGSC0003DMG400020341 | 17.5 kDa class I heat shock protein | F: TGGTCGTGATAAAGGTTGGAA  R: TTGACACCTAAAGAAATGTGCATC | -14.20 | -7.29 |
| PLD-47 | |  |  |  |  |
| 9. | PGSC0003DMG400027888 | Cysteine proteinase 3 | F: GAGAATCCGATCAGACAAGTCG  R: ACAAAGCGAGCAAAGGAGAG | 12.24 | 7.16 |
| 10. | PGSC0003DMG401004840 | Phosphatase | F: GATTTTCAAACATCCCCTCATGG  R: ACAGAAATCCCCGATTCCATC | -13.33 | -6.62 |

RT-qPCR analysis was performed using Kufri Bahar as control with test sample like RNA-seq.
