# Supplementary figures and images for: Screening of wild species and transcriptome profiling to identify differentially regulated genes in response to late blight resistance in potato
Source: Front Plant Sci. 2023 Jul 12;14:1212135. doi: 10.3389/fpls.2023.1212135 (PMC10368984; doi:10.3389/fpls.2023.1212135)

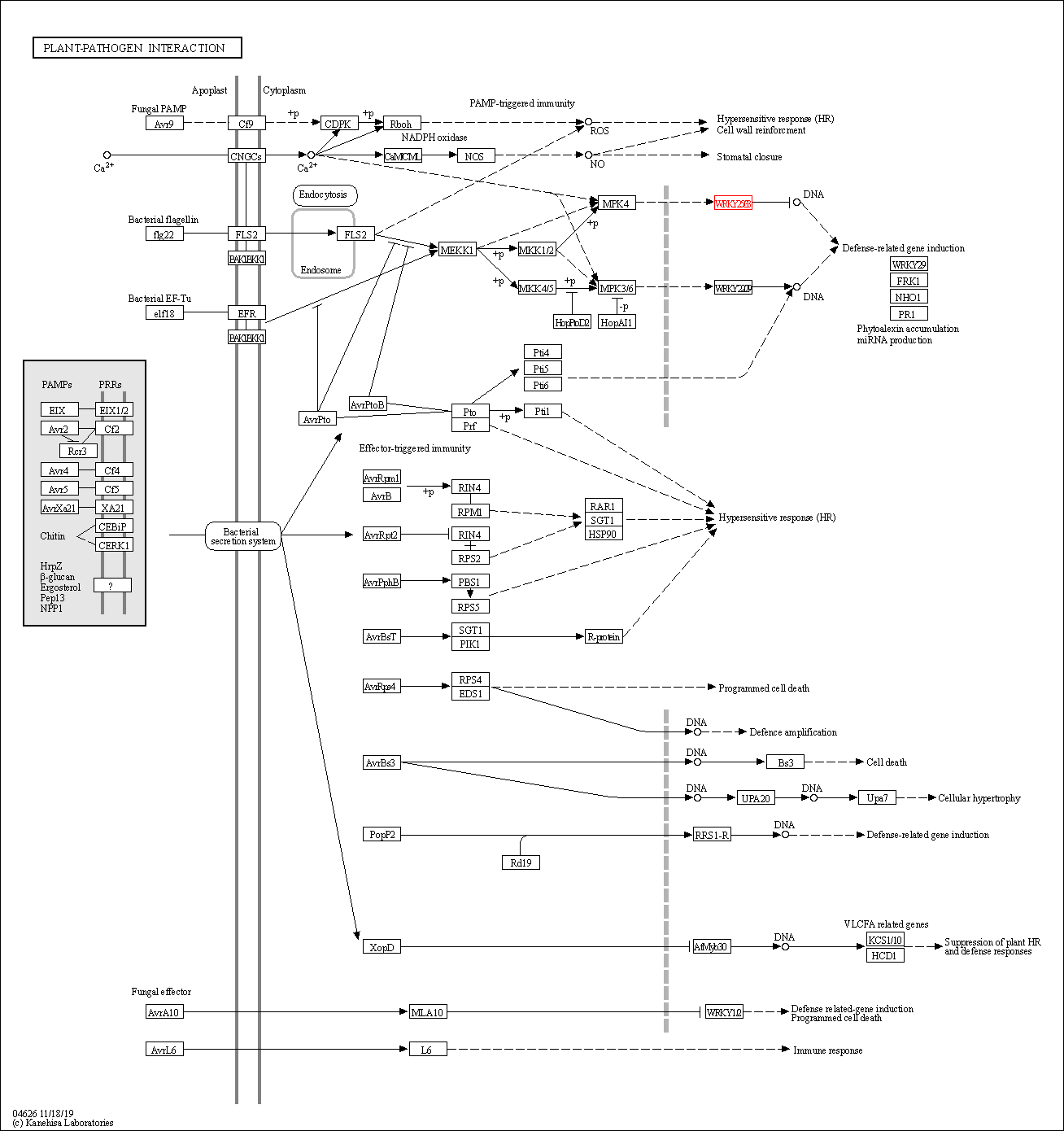

Supplement: Supplementary file 1 [file DataSheet_1.zip › Suppl. files_R1_10-6-23/Suppl. Fig. S6_map04626_plant pathogen inter..png]

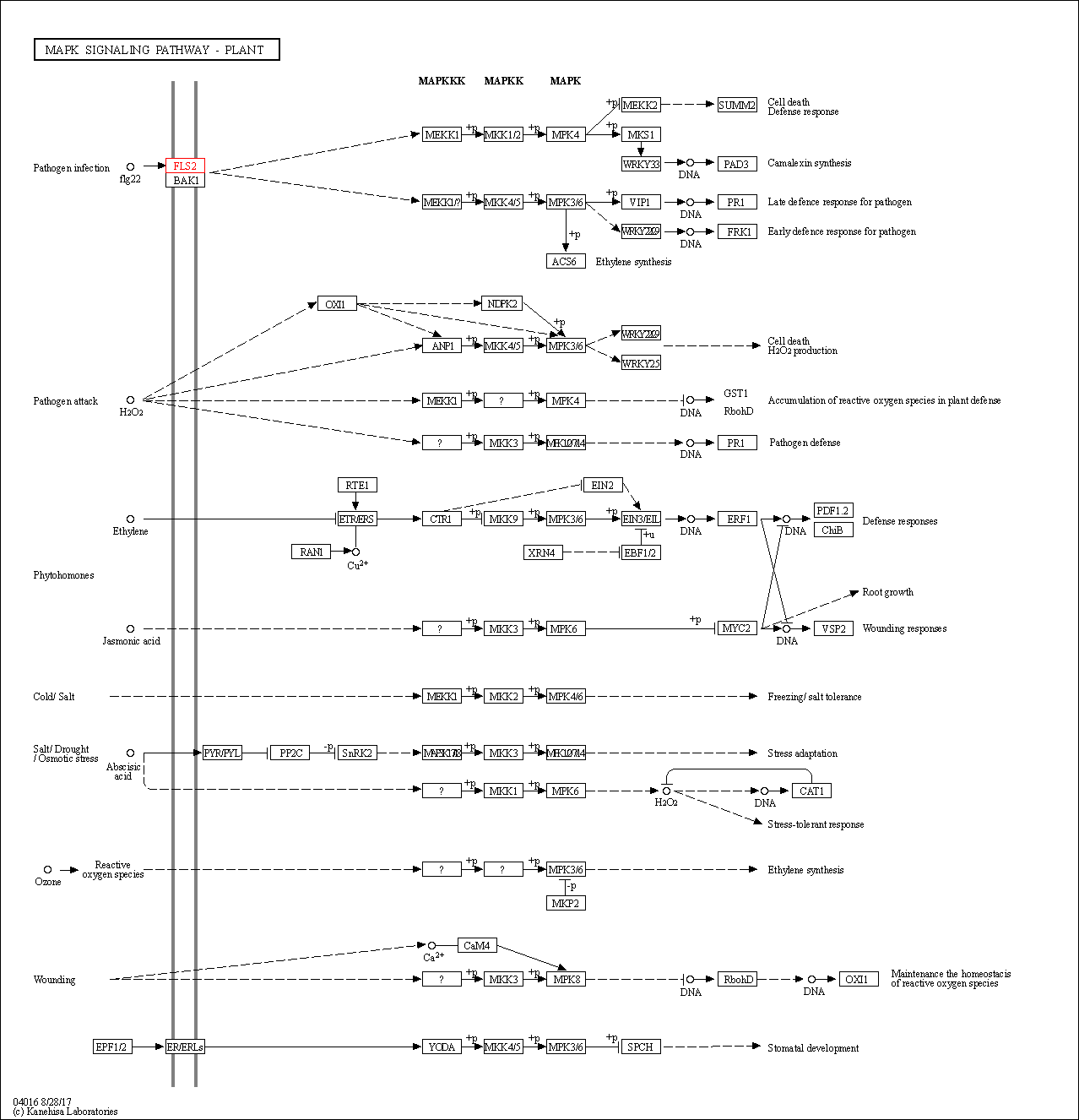

Supplement: Supplementary file 1 [file DataSheet_1.zip › Suppl. files_R1_10-6-23/Suppl. Fig. S7_map04016_Plant pathogen inter..png]
